# Reaction rebalancing: a novel approach to curating reaction databases

**DOI:** 10.1186/s13321-024-00875-4

**Published:** 2024-07-19

**Authors:** Tieu-Long Phan, Klaus Weinbauer, Thomas Gärtner, Daniel Merkle, Jakob L. Andersen, Rolf Fagerberg, Peter F. Stadler

**Affiliations:** 1 Bioinformatics Group, Department of Computer Science and Interdisciplinary Center for Bioinformatics and School for Embedded and Composite Artificial Intelligence (SECAI), Leipzig University, Härtelstraße 16-18, 04107 Leipzig, Germany; 2 Department of Mathematics and Computer Science, University of Southern Denmark, 5230 Odense M, Denmark; 3 Machine Learning Research Unit, TU Wien Informatics, Erzherzog-Johann-Platz 1 (FB02), A-1040 Wien, Austria; 4 Faculty of Technology, Bielefeld University, Postfach 100131, 33501 Bielefeld, Germany; 5 Max Planck Institute for Mathematics in the Sciences, Inselstraße 22, 04103 Leipzig, Germany; 6 Department of Theoretical Chemistry, University of Vienna, Währingerstraße 17, A-1090 Wien, Austria; 7 Facultad de Ciencias, Universidad National de Colombia, Bogotá, Colombia; 8 Center for non-coding RNA in Technology and Health, University of Copenhagen, Ridebanevej 9, 1870 Frederiksberg, Denmark; 9 Santa Fe Institute, 1399 Hyde Park Rd., Santa Fe, NM 87501 USA

**Keywords:** Reaction databases, Unbalanced reactions, Data curation, SynRBL, Rules, Maximum-common-subgraph

## Abstract

**Purpose:**

Reaction databases are a key resource for a wide variety of applications in computational chemistry and biochemistry, including Computer-aided Synthesis Planning (CASP) and the large-scale analysis of metabolic networks. The full potential of these resources can only be realized if datasets are accurate and complete. Missing co-reactants and co-products, i.e., unbalanced reactions, however, are the rule rather than the exception. The curation and correction of such incomplete entries is thus an urgent need.

**Methods:**

The SynRBL framework addresses this issue with a dual-strategy: a rule-based method for non-carbon compounds, using atomic symbols and counts for prediction, alongside a Maximum Common Subgraph (MCS)-based technique for carbon compounds, aimed at aligning reactants and products to infer missing entities.

**Results:**

The rule-based method exceeded 99% accuracy, while MCS-based accuracy varied from 81.19 to 99.33%, depending on reaction properties. Furthermore, an applicability domain and a machine learning scoring function were devised to quantify prediction confidence. The overall efficacy of this framework was delineated through its success rate and accuracy metrics, which spanned from 89.83 to 99.75% and 90.85 to 99.05%, respectively.

**Conclusion:**

The SynRBL framework offers a novel solution for recalibrating chemical reactions, significantly enhancing reaction completeness. With rigorous validation, it achieved groundbreaking accuracy in reaction rebalancing. This sets the stage for future improvement in particular of atom-atom mapping techniques as well as of downstream tasks such as automated synthesis planning.

**Scientific Contribution:**

SynRBL features a novel computational approach to correcting unbalanced entries in chemical reaction databases. By combining heuristic rules for inferring non-carbon compounds and common subgraph searches to address carbon unbalance, SynRBL successfully addresses most instances of this problem, which affects the majority of data in most large-scale resources. Compared to alternative solutions, SynRBL achieves a dramatic increase in both success rate and accurary, and provides the first freely available open source solution for this problem.

## Introduction

Large-scale reaction databases such as the United States Patent and Trademark Office (USPTO) database [1] and the commercial database Reaxys® [2] cataloge millions of chemical reactions and serve to enable data-driven approaches in chemistry. Reaxys®, hosting over 55 million manually curated reactions, has become a cornerstone for deploying deep-learning neural networks in retrosynthesis [3–7], robotic chemistry [8], and the determination of optimal reaction conditions [9].

USPTO is the largest public collection of chemical reactions, comprising more than 3 million entries mined from approximately 9 million US patents covering 1976 to 2016. Its impact on cheminformatics and synthetic chemistry is significant, and as a public resource, it has a particular impact on methods development. It plays a pivotal role in the advancement of reaction database analysis [10], forward [11–13] and backward [14] synthesis prediction, and yield prediction [15, 16]. The database has been instrumental also in reaction classification [17, 18], atom-atom mapping [19, 20], and synthesis rule clustering [21].

Despite the rapid advancements of databases, data quality remains a significant issue in particular for machine learning applications in chemistry [22]. A particularly serious problem is the omission of co-reactants or co-products. For example, less than 12% of the single step reactions in Reaxys®analyzed to study the exploration history of chemical space [23] were balanced. This problem has multiple roots, including historical and procedural practices. These deficiencies are attributed to the limitations of text mining, which struggles with the variability of publication formats [24], and to errors introduced during manual data curation [25].

Many data-driven applications therefore attempt to ignore the fact that many or most reactions are unbalanced and operate directly on such imperfect reaction data. This is in particular the case of atom-atom mapping methods. RXNMapper [20] and GraphormerMapper [26] apply machine learning for reaction mapping and atom embedding improvements, respectively, without directly addressing reaction imbalances. Jaworski’s rule-based atom-atom mapper [19], on the other hand, uses graph-theoretic considerations that introduce small molecules to achieve stoichiometric balance before atom correspondences are inferred. GraphormerMapper was reported to show enhanced performance on the Golden dataset of manually mapped and curated reactions [27]. Its efficacy on unbalanced reactions remains undocumented.

Several tools dedicated to balacing reactions have become available. CGRTools offers a rule-based method for rebalancing reactions by adding small molecules, which however has limited success in achieving perfect balance [28]. A hybrid workflow [29] combines ChemBalancer’s heuristic methods and ChemMLM’s machine learning to enhance molecule prediction. While ChemBalancer focuses on reaction completion, lacking precise accuracy metrics, ChemMLM shows promise with small molecules but struggles with complex structures [29].

The SynRBL framework for rebalancing reactions, which we introduce here, combines two methods: a rule-based approach for missing non-carbon compounds, i.e. compounds without carbon atoms like $\hbox {H}_{2}\hbox {O}$ or HCl, and a graph-theoretic approach for missing carbon structures. The rule-based method uses atomic symbols and counts to determine if reactions are balanced, decomposing molecules into ions to minimize redundancy and employing a search strategy that leverages a rule library to identify missing molecules.

For carbon compounds, we consider a maximum common subgraph (MCS) problem. This family of combinatorial optimization problems plays an important role in structural comparisons in chemistry and biology [30]. It underlies similarity searches vital to the preliminary phases of drug discovery, offering metrics for molecular structure similarity based on MCS dimensions, in alignment with the principle of similar properties [31, 32]. Beyond similarity assessment, MCS analysis is integral to clustering processes [33–35], the identification of matched molecular pairs [36], reaction mapping [37, 38], and the alignment of molecules [39]. MCS problems come in two flavors, both of which are NP-hard [40]. These two flavors are the maximum common induced subgraph (MCIS), which focuses on atom count, and the maximum common edge subgraph (MCES), which focuses on edge count. They give notable differences in the analyses of dissimilar molecules [41]. Our MCS-based approach targets carbon compound gaps and reactions beyond the rule-based method’s scope by aligning reactants and products to pinpoint and merge non-aligned segments, generating missing compounds. An iterative technique proceeding by overlapping molecules one at a time and isolating non-overlapping regions for efficient alignment in subsequent rounds is introduced to reduce computational costs.

## Method

### Notation and preliminaries

Every chemical reaction *r* can be written in the form

1 $$\begin{aligned} \sum _{i} s_{ir}^- X_i^{(q^-_{ir})} \rightarrow \sum _{j} s_{jr}^+ X_j^{(q^+_{jr})} \end{aligned}$$

where $s_{ir}^{-}\ge 0$ and $s_{jr}^{+}\ge 0$ are the stoichiometric coefficients of compounds $X_i$ and $X_j$ appearing as a reactant and as product, respectively. The superscripts $(q^{-}_{ir})$ and $(q^{+}_{jr})$ indicate the charge of the compounds $X_i$ and $X_j$ among the reactants and products, respectively. A molecule does not appear as a reactant or product if its stoichiometric coefficient vanishes, i.e., if $s_{ir}^{-}=0$ and $s_{jr}^{+}=0$, respectively. Since we consider only a single fixed reaction in the following, we drop the index *r* from here on.

Every compound $X_i$ has a well-defined composition expressed by its sum formula. We write $n_{ai}$ for the number of atoms of type *a* in compound *i*. The equilibrium of chemical reactions, grounded in the Law of Conservation of Mass by Antoine Lavoisier [42], stipulates that all reactions *r* are balanced in the sense that the total number $n^-_{ar}$ of atoms of type *a* in the reactants equals the total number $n^+_{ar}$ of atoms of type *a* in the products, i.e.,

2 $$\begin{aligned} n^{-}_{a} {:}{=}\sum _{i} n_{ai} s^{-}_{i} = \sum _{i} n_{ai} s^{+}_{i} =:n^{+}_{a} \end{aligned}$$

Similarly, the Law of Conservation of Charge ensures the constancy of total charge, crucial in redox and ionic reactions, i.e., it ensures that for every reaction

3 $$\begin{aligned} q^{-} {:}{=}\sum _{i} s^{-}_{i}q^{-}_{i} = \sum _{i} s^{+}_{i}q^{+}_{i} =:q^{+} \end{aligned}$$

In organic chemistry, carbon balancing (expressed as $n^-_{C}=n^+_{C}$), is essential for tracking carbon atoms in bond formations or cleavages, highlighting the significance of carbon atom accounting [43]. Balancing carbons is in practice more challenging because the imbalance is usually much larger compared to the atoms found in functional groups because larger organic molecules are not represented in the reaction data.

The task of reaction balancing can be expressed as follows. If a reaction is unbalanced, i.e., if $n^{-}_{a}\ne n^{+}_{a}$ for one or more atom types *a*, find a set of reactants $\{X_k^{(q^{-}_k)}\}$ and a set of products $\{X_l^{(q^{+}_l)}\}$ with non-zero stoichiometric coefficients $t_k^-$ and $t_l^+$ such that

4 $$\begin{aligned} n^{-}_{a} + \sum _{k} n_{ak} t^{-}_{k} = \sum _{l} n_{al} t^{+}_{l} + n^{+}_{a} \end{aligned}$$

holds for all atom types *a* and, likewise, the charges satisfy

5 $$\begin{aligned} q^{-} + \sum _{k} t^{-}_{k}q^{-}_{k} = \sum _{l} t^{+}_{l}q^{+}_{l} + q^{+} \end{aligned}$$

The practical complication is that (i) the set of possible compounds that may appear as additional reactants or products is too large for brute force enumeration, and (ii) even if this were possible, not all choices that formally might solve the problem are chemically plausible. To simplify the notation further, we can treat the charge as an additional formal “atom type” that may take on both positive and negative integer values, corresponding to positive and negative charges, respectively. This amounts to considering free electrons $\hbox {e}^{-}$ as a special compound. Moreover, we write $n_q^-$ and $n_q^+$ instead of $q^-$ and $q^+$ for the net charge in the following. Note that by convention a free electron $\hbox {e}^{-}$ corresponds to a charge of $-1$. We say that a reaction is (stoichiometrically) balanced if it satisfies Eqs. (4) and  (5), i.e., if the number of atoms of each type and the total charge is preserved between reactants and products. Otherwise, a reaction is stoichiometrically unbalanced.

In this contribution, we consider a particular kind of stoichiometrically imbalanced reaction: We assume that molecular sum formulas and/or structure formulas in a reaction entry are correct; therefore, the source of the observed stoichiometric imbalance is one or more missing compounds. The task of rebalancing a reaction, therefore, is defined here specifically as the inference of missing reactants or products to achieve stoichiometric balance. We emphasize that an entirely different approach would be required to handle a stoichiometric imbalance that originates from errors in the representation of compounds, resulting in erroneous sum formulas. In the remainder of this section, we describe two complementary strategies for rebalancing reactions as well as a workflow that integrates both approaches.

### Rule-based method

The essence of the rule-based approach lies in constructing a library of rules grounded in domain-specific knowledge. This library facilitates the imputation of missing compounds by addressing discrepancies between reactants and products. Subsequently, the missing elements are identified within the library and combined into the requisite compounds.

#### Representation of molecules and reactions

It is a common well-known issue that entries in reaction databases often omit one or more simple compounds such as $\hbox {H}_{2}\hbox {O}$, $\hbox {NH}_3$, and HCl.

To rebalance such incomplete reaction data, we developed a specialized rule library to systematically incorporate these missing elements utilizing the cheminformatics library RDKit 2023.9.4 [44]. To facilitate computations, we represent the sum formula of molecules as a dictionary.

$$\begin{aligned} \mathcal {D} {:}{=}\{ C_1: n_1, C_2: n_2, \ldots , C_{\ell }: n_{\ell }, Q: n_Q \} \end{aligned}$$

Here, each $C_a$, $1\le a\le l$, is an atomic symbol, i.e., $\hbox {H}$, $\hbox {O}$, or $\hbox {N}$, and $n_a \in \mathbb {N}$ is the number of atoms of type $C_a$ in the compound under consideration. We use the special symbol $\hbox {Q}$ to denote charge associated with the molecule. Recall that $n_Q \in \mathbb {Z}$ can be positive, negative, or zero.

The rule-based strategy is applied only to reactions that are carbon-balanced. The reason is that in organic reactions, the structure of the carbon backbone plays a key role, and thus, sum formulas are much less likely to be sufficient to completely describe the missing molecules. We also optimized our approach by considering the standard representation of ions in chemical equations, such as $\hbox {OH}^{-}$ and $\hbox {H}^{+}$, instead of $\hbox {NaOH}$ or $\hbox {HCl}$. To achieve this, we restructured our rule library to focus on elementary ions, enabling us to interpret compounds such as $\hbox {HCl}$ in terms of their constituent ions, $\hbox {H}^{+}$ and $\hbox {Cl}^{-}$. This refinement led to a more efficient and compact rule library, depicted in Table S3.

We denote by $\mathcal {D}^-$ and $\mathcal {D}^+$ the composition dictionaries of the sum of the molecular formulae of reactants and products, respectively. That is, $\mathcal {D}^-$ has entries of the form $C_a: n_a^-$, and $\mathcal {D}^+$ has entries $C_a: n_a^+$. The discrepancy between $\mathcal {D}^-$ and $\mathcal {D}^+$ is conveniently represented by two dictionaries $\Delta ^+$ with entries $C_a: n^+_a - n^-_a$ provided $n^+_a > n^-_a$, and $\Delta ^-$ with entries $C_a: n^-_a - n^+_a$ provided $n^+_a < n^-_a$. Thus $\Delta ^+$ accounts for the atoms only present in the products and $\Delta ^-$ accounts for the atoms only present in the reactants.

Based on the difference dictionaries $\Delta ^{\pm }$ we distinguish four cases:

- balanced if $$\Delta ^+ = \Delta ^- = \emptyset$$,
- reactant-dominated if $\Delta ^- \ne \emptyset$ and $\Delta ^+ = \emptyset$,
- product-dominated if $\Delta ^+ \ne \emptyset$ and $\Delta ^- = \emptyset$,
- both-sides if both $\Delta ^- \ne \emptyset$ and $\Delta ^+ \ne \emptyset$.

If only one of $\Delta ^-$ and $\Delta ^+$ has a non-charge entry, then the charge difference is accounted for in the same dictionary, while the other one is left empty. This is always possible since charges may be positive or negative. Instances of the both-sides case, i.e., instances with missing atoms in both reactants and products are not considered further here. They require a more sophisticated approach and are relegated to the MCS-based method in our current implementation.

Reactant-dominated and product-dominated cases are handled in the same manner. In the following, we denote by $\Delta$ the single non-empty difference dictionary.

For example, the database entry

$$\begin{aligned} {\hbox {CH}_{3}\hbox {COOH} + \hbox {C}_{2}\hbox {H}_{5}\hbox {OH} \rightarrow \hbox {CH}_{3}\hbox {COOC}_{2}\hbox {H}_{5}} \end{aligned}$$

yields the dictionaries $\mathcal {D}^- = \{\hbox {C}:4,\hbox {H}:10,\hbox {O}:3 \}$ and $\mathcal {D}^+ = \{\hbox {C}:4, \hbox {H}:8, \hbox {O}:2 \}$ for the reactants and product, respectively, and thus $\Delta ^-=\{\hbox {O}:1, \hbox {H}:2 \}$.

#### Molecular Imputation

For ease of presentation we assume $\Delta =\Delta ^-$, i.e., atoms are missing on the product side only. Otherwise, the role of reactants and products is interchanged.

We consider a set $\mathcal {R}$ of rules that explain (part of) the dictionary $\Delta$ in terms of molecules $X_k$ that are added to the product side. Our goal is to find a sequence of rule applications which stepwise reduce the difference dictionary $\Delta$ and collect a multiset *S* of molecules. Each $r\in \mathcal {R}$ is of the form $\hat{r}\rightsquigarrow X_r$, where $\hat{r}$ is a dictionary and $X_r$ is a corresponding molecule. The application of a rule changes $\Delta$ accordingly. Since our rules make use of simple ions, we allow arbitrary changes of charges. The rule

$$\begin{aligned} \{\hbox {O}:1, \hbox {H}:1, \hbox {Q}:-1 \} \rightsquigarrow {\hbox {OH}^{-}} \end{aligned}$$

applies to dictionary $\Delta =\{\hbox {O}:1,\hbox {H}:2\}$ by adding ${\hbox {OH}^{-}}$ to the products and updating the dictionary to $\Delta =\{\hbox {H}:1, \hbox {Q}:1 \}$. The resulting reaction is still unbalanced and reactant-dominated, hence another rule may apply.

If we reach $\Delta =\emptyset$, then adding *S* to the products balances the reaction. In practice, this can be achieved by a basic Depth-First Search (DFS), i.e., a recursive algorithm that explores the entire depth of the solution space before backtracking [45] as outlined in Algorithm 1. The search could, in principle, also be performed using another traversal strategy such as Breadth-First Search (BFS). Since the current implementation enumerates all optimal solutions, the performance of DFS and BFS is comparable. We opted for DFS because it lends itself more easily to heuristics that retrieve good solutions early in the search process and thus provides simple ways to reduce computational effort. A call to DFS($\Delta$, $\mathcal {R}$, $\emptyset$) either returns all (multi)sets of compounds *S* that balances the reaction and leaves an empty dictionary $\Delta$, or it terminates without output. In Algorithm 1, we write $\Delta \ominus \hat{r}$ for the dictionary obtained from $\Delta$ after it is modified by the application of a rule *r*.

**Algorithm 1:**
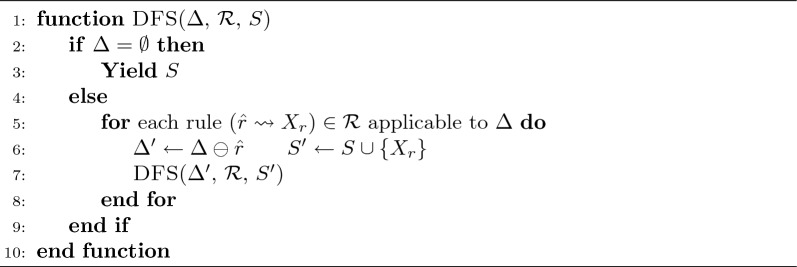
DFS-like rule application

The DFS algorithm yields all balancing solutions. These are passed on to the post-processing step (Sect. Post-processing). The list $\mathcal {R}$ of rules is applied in a fixed order that ensures that pattern size, defined as the number atoms in $\hat{r}$, is non-increasing. Thus, the search can be restricted to check only patterns with a valid length. One could use the fact that the dictionary obtained by the successful application of several rules is independent of the order in which these rules are applied. Keeping track of the rule *r* that was applied before DFS($\Delta ,\mathcal {R},S$) was called, it therefore suffices to disregard in the next recursion step all rules that appear before *r* in $\mathcal {R}$. Moreover, one could abandon a recursion step if its path length exceeds the best previously found solution. The latter modification however limits the scope of post-processing rules intended to remove chemically implausible solutions. Since simple DFS is already comparably fast and the search tree is usually quite shallow, such optimization is currently not implemented.

Continuing the example, after the first match, we may apply the rule $\{\hbox {H}:1,\hbox {Q}:1\}\rightsquigarrow {\hbox {H}^{+}}$, which leaves the dictionary $\Delta$ empty. The DFS function first gives $S=\{{\hbox {OH}^{-}},{\hbox {H}^{+}}\}$ and we arrive at the (chemically correct) balanced reaction

$$\begin{aligned} {\hbox {CH}_{3}\hbox {COOH} + \hbox {C}_{2}\hbox {H}_{5}\hbox {OH} \rightarrow \hbox {CH}_{3}\hbox {COOC}_{2}\hbox {H}_{5} + \hbox {OH}^{-} + \hbox {H}^{+}}. \end{aligned}$$

In general, there will be multiple solutions. Thus, continuing the DFS after it yields the first result turns it into an exhaustive search. The advantage of listing all solutions is that they can be evaluated, and an optimal solution can be identified. Here, we use the minimal number of rules as an optimization criterion. This favors matches of large partial dictionaries. When multiple solutions exhibit an equivalent minimal count of rules ascertained through the DFS algorithm, precedence is accorded to the solution that encompasses an ion in the set *S*. As an example, consider

$$\begin{aligned} {\hbox {CH}_{3}\hbox {CHBrCHBrCH}_{3} \rightarrow \hbox {CH}_{3}\hbox {C}\equiv \hbox {CCH}_{3}}. \end{aligned}$$

The missing compounds are detected on the product side with $\Delta =\{\hbox {H}:2,\hbox {Br}:2\}$, leading to two possible solutions: $S_1=\{2{\hbox {H}^{+}}, 2{\hbox {Br}^{-}}\}$ and $S_2=\{{\hbox {H}_{2}}, {\hbox {Br}_2}\}$. Here, the sorting function based on the minimal number of rules is ineffective since both solutions involve two rules. The presence of ions in the reaction serves as a decisive secondary criterion. SynRBL’s rule library gives preference to ions as the smallest units and thus favors $S_1$ in this scenario. This approach has its limitations when solutions depend on two criteria, and it may not be applicable to all reactions.

#### Post-processing

In some cases, the balancing of a reaction using DFS($\Delta$, $\mathcal {R}$, $\emptyset$) yields a formally correct solution that is chemically implausible. More precisely, *S* may contain one or more molecules that are at least unlikely to be the true reactants or products. In some cases, it is possible to find a more plausible rebalancing. Oxygen and halogens are typically formed via potent oxidizing agents. Hydrogen, on the other hand, is usually produced in reactions with alkali metals (e.g., lithium, sodium, potassium) or hydride compounds. Whether this is the case can be checked after DFS($\Delta$, $\mathcal {R}$, $\emptyset$) has successfully balanced the reaction. Currently, SynRBL considers only three post-processing rules:

(i) If a free halogen appears as a product, we assume that the solution is invalid and reject the completion.
(ii) If oxygen $\hbox {O}$ appears as a product, we add $\hbox {H}_2$ as a missing reactant and replace $\hbox {O}$ by $\hbox {H}_{2}\hbox {O}$ on the product side.
(iii) If hydrogen $\hbox {H}_{2}$ appears on the product side and there is neither an alkali metal nor a hydride among the reactant, we add $\hbox {O}$ to the reactants and replace $\hbox {H}_{2}$ by $\hbox {H}_{2}\hbox {O}$ on the product side.

The software is designed in a manner that makes it straightforward to extend this rule set.

#### Redox reaction refinement

Consider the reduction reaction involving the transformation of acetic acid into ethanol: ${\hbox {CH}_{3}\hbox {COOH} \rightarrow \hbox {C}_{2}\hbox {H}_{5}\hbox {OH}}$. The rule-based methodology aptly addressed this reaction by introducing two moles of hydrogen $\hbox {H}_{2}$ to the reactant side and one mole of water ($\hbox {H}_{2}\hbox {O}$) to the product side, thereby yielding the stoichiometric equation:

$$\begin{aligned} {\hbox {CH}_{3}\hbox {COOH} + 2\hbox {H}_{2} \rightarrow \hbox {C}_{2}\hbox {H}_{5}\hbox {OH} + \hbox {H}_{2}\hbox {O}} \end{aligned}$$

It is essential to acknowledge that the depicted reaction is not viable due to the insufficient reactivity of molecular hydrogen (${\hbox {H}_{2}}$) for the reduction of acetic acid. Typically, this reaction necessitates a suitable reducing agent, such as lithium aluminum hydride (${\hbox {LiAlH}_{4}}$). However, identifying and substituting the appropriate reducing agents can be problematic. Some chemists use a convention to simplify chemical notations where the reducing agent is represented as [$\hbox {H}$] without specifying the exact compound. Following this convention, we have updated the notation from molecular hydrogen (${\hbox {H}_{2}}$) to two single hydrogen atoms ($\hbox {H}$). This new representation indicates the presence of a reducing agent distinct from elemental hydrogen. Likewise, the depiction of molecular oxygen as ${\hbox {O}_{2}}$ has been revised to two single oxygen atoms ($\hbox {O}$), symbolizing its role as an oxidizing agent.

### MCS-based method

The fundamental principle of this methodology is the identification of the missing scaffold through the application of MCS techniques. Subsequently, chemical reaction rules are utilized to merge or modify these missing fragments, thereby resulting in the final compounds. In this section, we will explore in finer detail the various decision-making steps and the implementation of this method.

#### Determination of missing carbon compounds

Carbon-unbalanced reactions cannot be meaningfully handled at the level of sum formulas. Instead, it is necessary to make use of the structures of reactant and product molecules. To this end, we represent both the reactants and the products of a reaction as graphs whose connected components are the molecules. In these graphs, vertices are labeled by atom types and edges correspond to chemical bonds, annotated by their bond type. Since reactions with carbon atoms missing on the reactant side are treated in the same way as reactions with missing carbon on the product side, we fix the notation as follows:

Let *X* and *Y* be the graphs with the larger and smaller number of carbons, respectively. Moreover, we write $\mathcal {X}=\{X_1,X_2,\dots ,X_{k}\}$ for the set of connected components of *X*. Assuming that all missing carbons belong to one connected compound $Y_*$ missing on the *Y*-side of the reaction, we can conclude that $Y_*$ is in essence a part of some $X_i$. In order to identify this part, we compute, for each $X_i\in \mathcal {X}$, a maximum connected common subgraph $M_i={{\,\textrm{MCS}\,}}(X_i,Y)$. There are several choices for the exact definition of the function ${{\,\textrm{MCS}\,}}(\,.\,)$, which we will discuss in more detail below. For the moment we only require that the subgraph $M_i$ is connected and that ${{\,\textrm{MCS}\,}}(\,.\,)$ defines an injective map of the vertex set $V(M_i)$ into $V(X_i)$ and *V*(*Y*) where each vertex in *V*(*Y*) is only mapped once. We can therefore identify the vertices of $M_i$ with a subset of the vertices of $X_i$ and, by a slight abuse of notation, simply write $V(M_i)\subseteq V(X_i)$. This, in turn, specifies a (bipartite) matching between vertices of $X_i$ and *Y* that correspond to the same vertex of $M_i$. In chemical terms, this matching is a partial atom-atom map between $X_i$ any *Y* and thus also between *X* and *Y*. To characterize the part of $X_i$ that does not match *Y* in more detail, we consider the subgraph $A_i{:}{=}X_i[V(X_i)\setminus V(M_i)]$ of $X_i$ induced by the unmatched vertices. Moreover, let $B_i$ be the edge cut between $V(A_i)$ and $V(M_i)$ in $X_i$. In chemical terms, $B_i$ denotes the bonds that separate $M_i$ and $A_i$ and thus were broken (or formed) by the reaction. A vertex in $A_i$ is said to be a boundary vertex if it is incident to a cut edge $e\in B_i$.

Denote by $\mathcal {A} {:}{=}\{ (A_i,B_i)| X_i\in \mathcal {X}\}$ the set of auxiliary graphs together with their separating edge cuts. We shall refer to these as fragments. By construction, $\mathcal {A}$ contains the relevant information on the missing compounds because the union $\bigcup _i V(A_i)$ is the set of missing atoms, and the $B_i$ are bonds on $X_i$ that are broken in order to obtain *Y*. The task at hand, therefore, is to “recombine” the $(A_i,B_i)$ in a way that recovers the missing compound(s) $Y_*$. To this end, we again pursue a rule-based approach. We consider two types of rules:

**Fig. 1:**
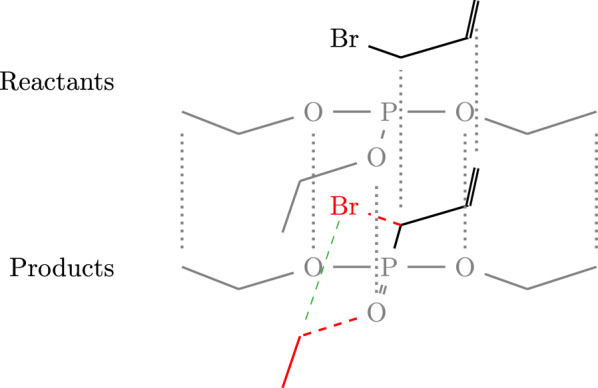
In this example two fragments (shown in red) remain unmatched: $\hbox {Br}$ with a single bond as cut, and an ethyl group also with a single bond. The cut edges of the fragments are shown as dashed red lines. A merge rule inserts a single bond (dashed green) connecting the end-points of the cut edges

*Merge rules* encode conditions for the insertion of edges between two boundary vertices, $u\in V(A_i)$ and $v\in V(A_j)$ located in distinct fragments, see Fig. 1. These rules depend on the specific boundary configuration, i.e., the chemical context of the two boundary atoms *u* and *v*. The application of a merge rule not only inserts a bond (labeled edge) between *u* and *v*, but also removes the respective cut edges incident to *u* and *v* from $B_i$ and $B_j$, respectively. Thus only one merge rule is applied for each boundary. The boundaries are then considered resolved in the chemical domain. Moreover, open boundaries on the same compound are never merged with each other. Hence, this step always needs at least two compounds. If only one is available, expand rules are applied first to add the missing second fragment. A collection of merge rules is provided as configuration file and can easily be extended or modified in SynBRL. Table S1 in the supplementary lists the currently implemented merge rules. The alignment and imputation on a simple example are depicted in Fig. 1.

*Expand rules* are used to add nodes to the molecular graph based on the boundary configuration of unmatched fragments. More precisely, they can add fragments with boundaries to $\mathcal {A}$ depending on what is needed for unresolvable cut edges. This is in particular the case if $\mathcal {A}$ comprises only a single fragment (*A*, *B*). The idea of the expand rules is to add additional atoms such that cut edges that do not have a counterpart in another fragment are “saturated”. Technically, however, an expand rule only adds the required atom, and the actual bond is then formed by a merge rule. Expand rules are also specified in a configuration file. Table S2 in the supplementary lists the currently implemented merge rules.

Each application of a merge step reduces the number of cut edges in the fragment set $\mathcal {A}$. Repeated rule application either terminates prematurely with no further applicable rule, or it succeeds replacing all cut-edges, thus resulting in a graph *Z* without remaining boundary vertices. By construction, the reaction $X\rightarrow Y \cup Z$ is now carbon balanced. It is not balanced in general. Note that the expand steps have added additional non-carbon atoms.

**Fig. 2:**
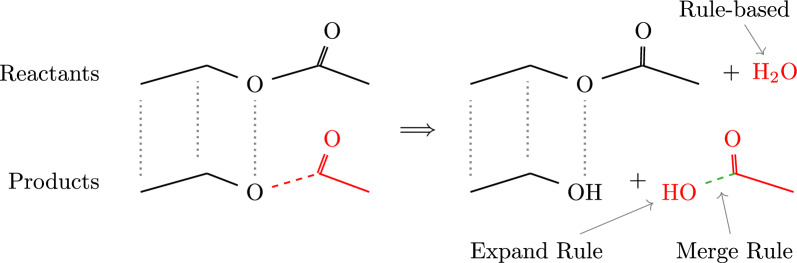
Visualization of MCS-based rebalancing, on the reaction of ethyl acetate to ethanol. The first step of rebalancing is identifying the best alignment of product and reactant graphs (dotted black lines) corresponding to a maximum common subgraph. Subtracting the common structure leaves the raw missing compound, here the acetaldehyde shown in red on the left side. The dashed red line indicates the broken connection between the missing compound and the maximum common subgraph. Adding the missing compound may not be sufficient to obtain a chemically reasonable result. Thus a set of expand and merge rules are applied to fix the raw structure. In this example acetaldehyde is implausible. The lack of a second reactant triggers an expand rule that appends necessary oxygen, which is then attached to the new compound by a merge rule. In this case the merge rule creates a single bond between the two fragements, depicted by the dashed green line. This results in acetic acid as product. A valid solution is carbon balanced but not necessarily balanced after this step. The Rule-based method is therefore used to impute the missing water on the reactant side

In practice, most carbon unbalanced reactions are missing a structure at the product side of the reaction. Hence, the methodology focuses on reactant-dominant reactions. In principle, it can be applied to product-dominant reactions as well. However, imputing a missing reactant is more challenging than finding a missing product. A single reaction equation can often contain multiple reaction steps, leading to multiple equally correct intermediate compounds that could be added to the reactants to form a balanced reaction. Since these cases are of minor practical relevance, we have not attempted to formulate specific rules for product-dominant reactions.

Figure 2 shows a simple de-esterification as an example. Here, only one missing fragment is detected. Because the carbon-oxygen bond is part of an ester group, an expand rule adds the missing oxygen atom to the reaction. In the second step, a merge rule connects this oxygen with a single bond to the open boundary on the identified fragment, creating the missing acetic acid. The resulting reaction is carbon balanced but unbalanced overall. The rule-based method described in Sect. Rule-based method is now applicable to add the missing water molecule to the reactants.

#### Computing maximum common molecular subgraphs

Maximum common subgraph (MCS) problems come in different variants. Both the maximum common induced subgraph (MCIS) problem and the maximum common edge subgraph (MCES) problem, as well as their restrictions to connected common subgraphs, are NP-hard [40]. Nevertheless, they can be solved efficiently for small pairs, and thus also for molecules. However, none of the variants of the combinatorial optimization problem is guaranteed to identify the “chemically correct” common subgraph, i.e., the one that correctly identifies all bonds that change during a chemical reaction.

**Fig. 3:**
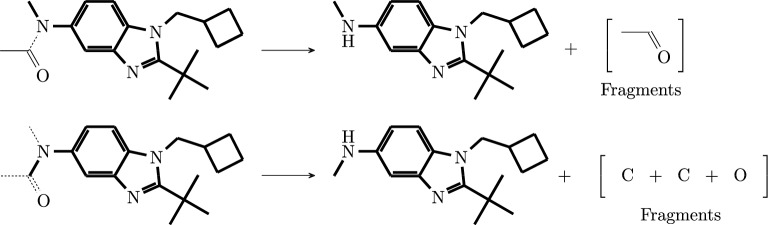
Ambiguity of maximum common subgraphs. The bold black lines highlight the MCS between product and reactant molecule. The MCS has two distinct isomorphisms in the reactant. The alignment in the first row creates one fragment, and the isomorphic alignment in the second row causes three fragments. Dotted lines indicate the broken bonds resulting from the product alignment. The fragments visualize the remaining structure after subtracting the MCS from the reactant graph

While the size of an MCS is uniquely defined, neither the common subgraph nor its embedding is unique in general. In the example in Fig. 3 the subgraph isomorphism for the bold black subgraph is not unique. This is a well-known issue for the construction of atom-atom mapping tools. These ambiguities are not easily resolved because the combinatorial MCS problems operate on graphs rather than a more detailed model of the molecules that encompasses also e.g. hybridization or partial charges.

In order to improve over the application of any one particular problem variant or algorithm, SynRBL resorts to the heuristics implemented in RDKit [44] and computes several alternative variants: MCIS is addressed using the Fragment Matching and Compound Similarity (FMCS) [46], while the Rascal algorithm [47], as implemented in the RDKit library, is used to solve the MCES problem. Moreover, an ensemble method that amalgamates outcomes from five distinct configurations is used, detailed in Table 1. Each of these specifies additional constraints on the matches allowed in the corresponding MCIS or MCES variant. Both the RingMatchesRingOnly and the CompleteRingsOnly ensure that atoms in rings match atoms in rings only. In graph-theoretical terms this corresponds to singling out the vertices in non-trivial 2-connected components. With the latter option, rings must be matched completely. In addition, bond order (treated as edge label) can be used as a constraint to prohibit the matching on single and double bonds.

**Table 1:** MCS configuration

Configuration	1	2	3	4	5
RingMatchesRingOnly	True	True	False	False	–
CompleteRingsOnly	True	True	False	False	–
Ignore Bond Order	True	False	True	False	–
Algorithm	FMCS	FMCS	FMCS	FMCS	RASCAL

In order to deal with alternative embeddings of the MCS, we enumerate all maximal solutions of ${{\,\textrm{MCS}\,}}(X_i,Y)$ and identify the solutions that minimize the number of fragments resulting from the removal of the common subgraph. This choice is grounded in the Principle of Minimum Chemical Distance (PMCD), which states that reactions tend to involve a minimal number of bond changes [48]. The criterion aligns well with the selection of the substructures that results in the fewest number of fragments, which is equivalent to the minimal number of bond changes during the reaction. In the example in Fig. 3, one isomorphism corresponds to the disruption of the amide bond CO-N, thereby producing one additional fragment. The alternative embedding of the same common subgraph implies breaking bonds containing the amine bond ${\hbox {CH}_{3}-\hbox {N}}$, resulting in three additional fragments. Hence, we choose the former embedding.

In order to keep the computational costs low, we do not compute ${{\,\textrm{MCS}\,}}(X,Y)$ directly, but instead use an iterative approach that successively aligns the components $X_i\in \mathcal {X}$ and removes the matched vertices from *Y*. More precisely, for each $X_i\in \mathcal {X}$ we compute ${{\,\textrm{MCS}\,}}(X_i,Y^{(i-1)}))$ and construct $Y^{(i)}$ by removing all matched vertices from $Y^{(i-1)}$. To do this efficiently, we sort $\mathcal {X}$ in order of decreasing number of vertices in the connected components. As part of each evaluation of ${{\,\textrm{MCS}\,}}(X_i,Y^{(i-1)}))$ we also keep track of the cut edges between the matched and unmatched vertices, i.e., the broken bonds, which in particular allows us to compute the $(A_i,B_i)$ from the iterative MCS approach.

**Fig. 4:**
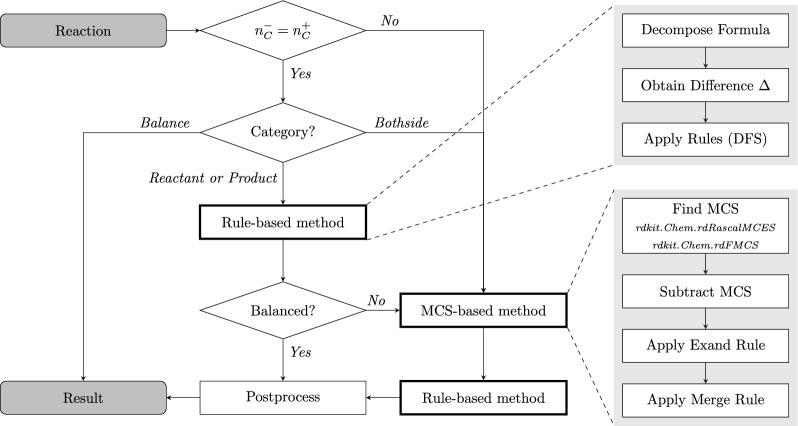
Simplified overview of the functional process in SynRBL. The rule-based method is applied if the reaction is carbon-balanced but otherwise unbalanced in either the reactant or the product side. The MCS-based method is used if both sides are unbalanced, the rule-based method fails, or the reaction has a carbon imbalance in the first place. The output is either the balanced reaction if the method is successful or the unmodified input in case SynRBL can not find a solution. The Rule-based and MCS-based method blocks abstract the respective procedures for the sake of readability. Supplementary Fig. S1 and Fig. S2 contain a more detailed in-depth view on these blocks with example data

### Interaction of the two methods

The rule-based method offers efficient solutions for non-carbon compounds, whereas the MCS-based approach focuses on subgraphs to find missing carbon structures. Identifying the optimal common subgraph is computationally intensive, making the MCS-based method less suitable for non-carbon compounds. Consequently, applying the two methods complementarily, each to their respective optimal scenarios, enhances overall efficiency: the rule-based approach for non-carbon compounds and the MCS-based method for situations where subgraph analysis is advantageous. The overall framework is summarized in Fig. 4. Reactions identified as bothside have a non-carbon imbalance on the reactant and product side. These cases are not solvable by the rule-based method and are hence subject to the MCS-based method. Both methods utilize functions from RDKit [44]. Either for parsing reaction SMILES or handling the molecular graph representation in the MCS-based method.

Just like the rule-based method, the MCS-based method can only solve some imbalances. More precisely, the approach depends on the identification of the chemically correct MCS. The method outlined above, in particular, cannot handle rearrangement reactions or ring-formations. We shall return to this point in more detail, see Sect. MCS-based method below. The MCS-based method also tends to fail if too many compounds or boundaries are found, the number of boundaries does not match, or the reaction is not carbon balanced afterwards, e.g., because not all carbon atoms in *Y* are covered by MCS matches. On the other hand, if a solution is found, the confidence is high that the result is in fact correct.

### Datasets and benchmarking

SynRBL is not trained on any specific dataset but leverages basic chemical knowledge to inform its rule set. In order to assess its performance we use three widely used public data collections: (i) an open-access tailored for CASP that incorporates the Golden dataset [27], (ii) Jaworski’s dataset [19], and (iii) the USPTO_50k collection [5]. The latter contains more than 50,000 reactions. We extracted a representative subset comprising only unbalanced reactions and selected validation datasets based on three different strategies, resulting in the following three datasets. The USPTO Random Class dataset (Urnd) was chosen to utilize a stratified sampling method across ten varied chemical reaction classes. Additionally, the USPTO Different dataset (Udiff) was selected employing a similar stratified strategy, albeit with $\Delta$, the difference in the dictionaries representing reactants and products, to ensure a comprehensive representation of the diversity in molecular formulas between reactants and products. The USPTO Unbalance Class (Uunb) was selected by randomly choosing from reactions classified as solved or unsolved by the rule-based method. This selection provides insights into carbon and non-carbon imbalances within the chosen reaction classes. To ensure reproducibility, the random seed was set to a fixed value ($\text {seed value} = 42$) for all random selection processes. The datasets are summarized in Table 2.

**Table 2:** Composition of validation datasets in different categories

Dataset	Reactions	${C}_{{unb}}$	Balance	Unbalance
Golden	1851	729	209	913
Jaworski	637	116	302	219
Urnd	803	328	0	475
Udiff	1589	355	0	1234
Uunb	540	257	0	283
Total	5420	1785	511	3124

In order to benchmark SynRBL we evaluated (1) success of the algorithm, defined as the fraction of (unbalanced) instances for which SynRBL proposed a balanced reaction, and (2) accuracy, the fraction of proposed solutions for the rebalancing problem that are (chemically) correct.

### Estimating prediction confidence

The results for the five datasets mentioned in Table 2 were checked manually by TLP, the first author, an experienced chemist. We reviewed all reactions to determine their chemical validity, typically focusing on whether the reaction center or bond changes were valid. The results presented in Sect. Results and discussion provide a good indicator of how many of the imputations should be correct. However, validating individual outcomes necessitates the expertise of a domain specialist. Predicting a confidence for results from the MCS-based method can be used to filter out potentially wrong imputations and increase the accuracy of the method. We observed that the accuracy strongly depends on the complexity of the reaction center, for example on the number of bonds involved in the reaction. We therefore developed a machine learning model using the XGBoost algorithm [49] (version 2.0.3) to predict a confidence value for our imputations based on the reaction properties illustrated in Table 3. A total of 2275 reactions were rebalanced using the MCS-based method from the five distinct datasets described previously. Each reaction was manually classified as either correctly rebalanced (1) or incorrectly rebalanced (0). We then stratified-split these reactions into two groups: 1,820 reactions (80%) were allocated to the training set, and 455 reactions (20%) were designated for the testing set, ensuring a representative distribution of outcomes in both sets.

**Table 3:** Features for analysis

Features	Description
*total_carbons*	The total count of carbon atoms present in the reaction
*total_bonds*	The aggregate number of chemical bonds in the reaction
*total_rings*	The total count of ring structures within the reaction
*fragment_count*	The total number of distinct fragments or molecules present in the reaction
*carbon_difference*	The discrepancy in the number of carbon atoms between reactants and products
*num_boundary*	The count of boundary atom (reaction center) identified by MCS-based method
*Bond Changes*	The maximum count of bonds formed in products or broken in reactants, a feature that requires manual extraction
*bond_change_merge*	The net change in the number of bonds between reactants and products post-MCS process
*ring_change_merge*	The net change in the number of rings between reactants and products post-MCS process

As part of the inspection, we manually counted the bond changes of all 2275 reactions. Since this is not feasible for any large-scale analysis, we used the feature “Bond Changes” only in the initial analysis and then leveraged other, easily calculable, features to train the confidence model.

To optimize the performance of the model in light of the imbalanced dataset, where the number of correct and incorrect solutions varies significantly, we employ the SMOTETomek algorithm [50] from imblearn 0.12.0 [51]. This technique combines the Synthetic Minority Over-sampling Technique (SMOTE) with Tomek links to effectively balance the dataset, thereby enhancing the predictive accuracy of our model. We applied SMOTETomek exclusively to the training set, increasing the number of data points from 1,820 to 3,024. Subsequently, XGBoost was trained on this augmented dataset. The performance of the model was validated on the test set, which retained its original size of 455 reactions.

## Results and discussion

### Rule-based method

The rule-based approach of Sect. Rule-based method is applicable on the reactions with missing compounds among either the reactants or the products, with the stipulation that the carbon must be balanced. This method yields a good success rate ranging from 89.60% to 99.69% on our five benchmarking sets. It reaches a rather remarkable accuracy level of up to 99.91% on the successful instances. These results are summarized in Fig. 6A below.

**Fig. 5:**
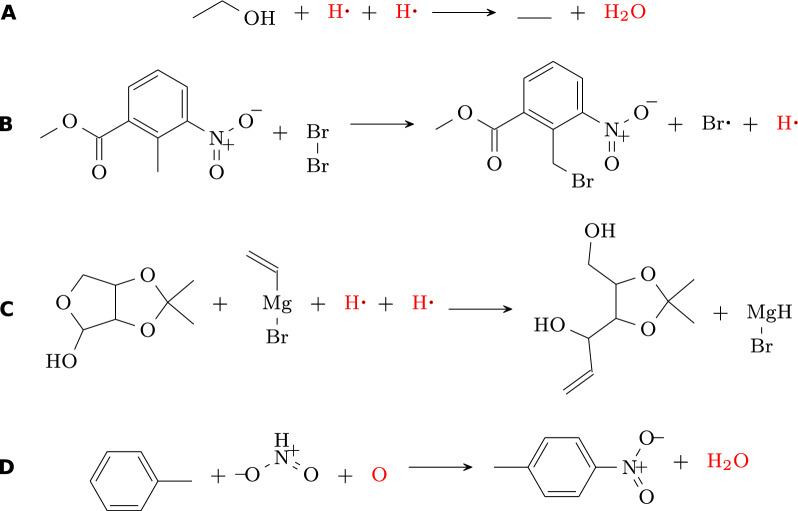
Examples for incorrect imputations with the rule-based method. Original database entries are shown in black, imputed compounds in red. **A** An erroneous reaction from USPTO, with $\Delta = \{{O}:1,{Q}:0\}$, representing a sequence of dehydration and reduction reactions. **B** A correctly rebalanced reaction from Jaworski dataset that remains uncertain due to the presence of Hydrogen on the product side. **C** False imputation in Jaworski dataset where the product is mistakenly standardized as RMgH instead of $\hbox {RMg}^{+}$. **D** An error in the rebalanced reaction in Golden dataset, due to ${\hbox {HNO}_{2}}$ being incorrectly identified instead of ${\hbox {HNO}_{3}}$ on the reactant side

*Analysis of incorrect predictions*

A careful inspection of invalid imputations revealed some systematic problems associated with specific datasets. Applied to data derived from the USPTO database (Urnd, Udiff, Uunb) the rule base method produced uncertain predictions associated when $\{{O}:1, {Q}:0\}$ being on the reactant side during rule application. Consider, for example the conversion of ethanol to ethane in Fig. 5A, which is usually performed by dehydration and subsequent hydrogenation or by application of hydroiodic acid HI.

In the Jaworski dataset, two reactions were flagged as uncertain or invalid. The first instance involved the presence of hydrogen in the product without alkali metals or hydrides. This anomaly was traced back to a precursor reaction involving a bromine radical Br∙, from which the generation of a hydrogen radical H∙ is incorrectly inferred. Instead of separate radicals, the formation of hydrogen bromide HBr is expected, see Fig. 5B. Further scrutiny revealed inaccuracies e.g. in Grignard Reactions, where the product was incorrectly identified as RMgH instead of ${\hbox {RMg}^{+}}$. This error could be attributed to the standardized procedures of the original database, which led to the improper imputation of hydrogen on the reactant side. The appropriate correction would be the addition of ${\hbox {H}^{+}}$ to the reactant side and $\hbox {RMg}^{+}$ to the product side, Fig. 5C.

In the Golden dataset we found 22 reactions with ambiguous status due to invalid reactants. Notably, the formation of nitrobenzene from benzene (id_481, Fig. 5D), erroneously specified nitrous acid ${\hbox {HNO}_{2}}$ instead of nitric acid ${\hbox {HNO}_{3}}$ as the reagent. The invalid reactions are enumerated in a dedicated supplementary file. A recurrent pattern observed in these reactions is that the rule-based method infers a singular oxygen O to be added to the reactant side.

Overall, however, the rule-based method rarely produces chemically incorrect or questionable imputations, at least when reactants and products are chemically accurate. The presence of isolated O or H in the prediction, on the other hand, appears to serve as an indicator for errors in the database entry.

**Fig. 6:**
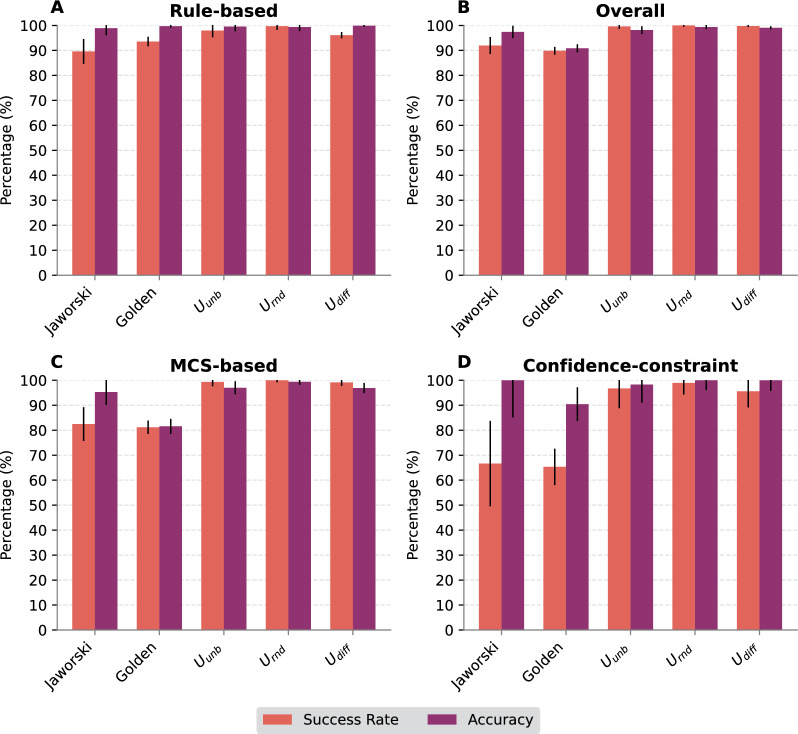
Validation results for the rule-based method (**A**), the entire framework (**B**), MCS-based method (**C**), and the MCS-based method with an applied confidence threshold of $50\%$ (**D**). Comparing (**C**) and (**D**) shows the tradeoff in success rate for higher accuracy when thresholding the predicted confidence. Because validation was only done on data that was not used in training (20% of the data), (**D**) has noticeably larger uncertainty margins

The rule-based approach is challenging with respect to computational cost if the compounds contain a larger number of carbon atoms and, in particular, if the number of carbon isomers becomes large. We also note that the method has difficulties with carbon-imbalaneced compounds in general. For example, in the reaction ${\hbox {CH}_{3}\hbox {COOC}_{2}\hbox {H}_{5} \rightarrow \hbox {CH}_{3}\hbox {COOH}}$, a naive solution might suggest adding ethylene ${\hbox {C}_{2}\hbox {H}_{4}}$ to balance the product side. The correct solutions, however, is to add water H$_{2}$O to the reactants and ethanol ${\hbox {C}_{2}\hbox {H}_{5}\hbox {OH}}$ to the products. Since such examples are abundant, we do not apply the rule-based method to carbon-imbalanced reactions.

### MCS-based method

**Fig. 7:**
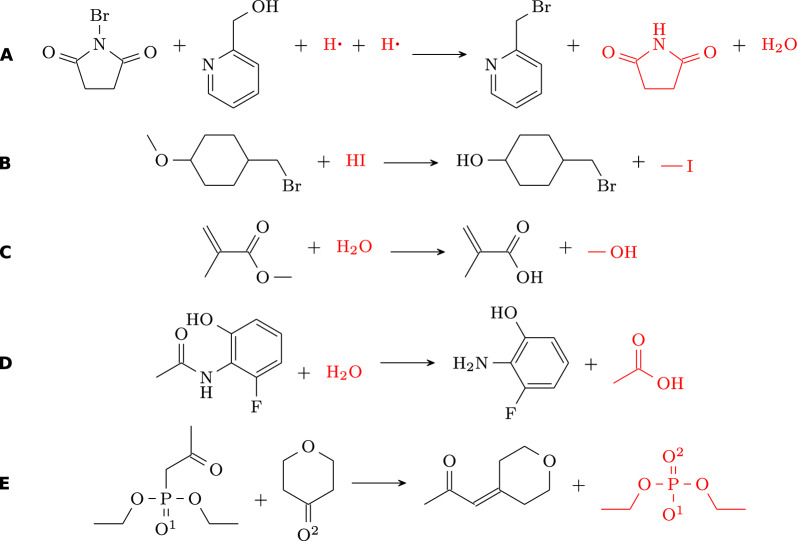
Some examples of reactions solved by the MCS-based method showcasing different merge and expand rules. Database entries are shown in black, imputed compounds in red. **A** Append compounds without forming a bond. **B** Append and merge I on Ether break. **C** Append and merge O on Ether break. **D** Append and merge O on Amide break. **E** Create new double bond with P. The double bond between ${\hbox {O}}^1$ and P in the reactant is changed to a single bond in the product and the oxygen ${\hbox {O}}^{2}$ from the oxan-4-one creates a double bond with P

The MCS-based method succeeds in 81% (Golden dataset) to 100% (Urnd of the test cases, see Fig. 6C and Supplementary Table S4. Fig. 7 depicts some reactions that were successfully balanced by the MCS-based method. It showcases the application of a list of different expand and merge rules. In contrast to the rule-based approach, the prediction accuracy on successful cases is not fully satisfactory on all test sets. While the predictions are close to perfect on the USPTO-based datasets, and about 95% for the Jaworski’s data, only about 80% are achieved on the Golden set. The differences in success rates between the datasets can be attributed primarily to differences in the frequency of reactions that cannot be balanced by the MCS-based approach, in particular rearrangement reactions, ring-formations, or complex reactions with many compounds.

*Analysis of Incorrect predictions*

**Fig. 8:**
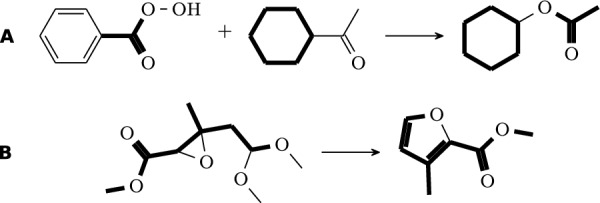
Two examples that are not solvable by the MCS-based method. The MCS is not meaningful for these types of reactions. **A** Example for an unsolvable oxidation and rearrangement reaction. **B** Example for an unsolvable ring-closing reaction. Bold lines indicate the identified MCS

Incorrect predictions arise in particular for complex reactions, and especially with multi-step reactions. Fig. 8 illustrated examples of a ring-forming reaction and a rearrangement reaction where the MCS-based approach fails to identify a valid solution. In Fig. 8A, the Baeyer-Villiger Oxidation involves multiple elementary steps or mechanisms. The MCS-based method is unable to identify a solution for this type of transformation. The structure highlighted as the MCS search result, particularly in Fig. 8B, exhibits four boundaries, indicating an erroneous outcome from the MCS-based method. Such reactions, not amendable by this method, are left unbalanced and represent a limitation of our approach in its current form.

In order to better understand other factors contributing to incorrect predictions, we investigated the influence of different features on the accuracy—see also Sect. Estimating prediction confidence. Not surprisingly, the accuracy decreases with indicators for the “complexity” of the reaction, particularly with the inferred number of broken/formed bonds, the total number of substances in the reaction, and the number of boundaries. A similar trend is found for the number of different bonds and cycles after graph merging. In contrast, the performance does not depend systematically on the carbon imbalance $|\hbox {n}^{+}_{\hbox {C}}-n^{-}_{\hbox {C}}|$. The total number of compounds in a reaction exceeds 6 only in some entries in the Golden dataset since it also reports catalysts and solvents. This suggests that the performance declines with more fragments due to potential substance-matching misalignments. In some cases, no boundaries were detected in the MCS step. The lack of accuracy in the absence of a boundary strongly suggests exiting without success if no boundary is found, since the result is almost always wrong anyway. The details of this exploratory data analysis are summarized in Supplementary Fig. S4.

**Fig. 9:**
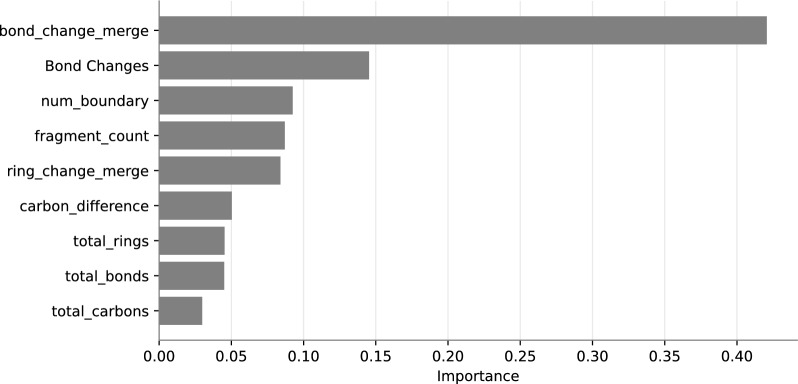
Feature importance analysis provides a detailed visualization of various factors influencing the precision of the MCS-based method

In order to understand the factors influencing accuracy in more detail we performed a feature importance study summarized in Fig. 9A. The feature importance is the average gain, i. e., the relative contribution of each feature for a given prediction over all targets. In line with the exploratory analysis described above, we observed that the total number of carbons, bonds, rings, and the difference in carbon content within the reaction does not significantly influence the performance of SynRBL. Surprisingly, the disparity in the bond count after graph merging emerged as the most impact factor, surpassing even the number of bond changes in predictive power. In order to investigate the interplay between the most informative factors, we also considered the co-occurances of the number of different bonds after merging, the number of different rings after merging, the count of boundaries detected, and total number of compounds, see Fig. S5.

Taken together, this analysis establishes parameters for which we can expect reliable rebalancing results: bond changes after merging should not exceed three; ring changes should be fewer than two; reactions should not involve more than four molecules, and only one or two boundaries should be detected.

As a more quantitative approach, we devised a scoring function that summarizes the feature analysis and allows estimating the confidence level of our predictions, see Sect. Estimating prediction confidence. The performance of our model is detailed in Supplemental Fig. S6, showcasing strong predictive capabilities with an F1-score (micro) of 0.92, an AUC of 0.94, and an AP of 0.81. Using a confidence threshold of 50%, leads to the expected increase in accuracy of the MCS-based predictions for both Jaworski’s dataset and the Golden dataset, at a moderate decline in success rate, see Fig. 6D. This observation underscores the robustness of the method in enhancing prediction reliability through the strategic application of a confidence threshold.

### Performance of the combination of rule-base and MCS-base components

The interplay of the rule-based and MCS-based methods described in Sect. Interaction of the two methods results in a satisfactory performance of the SynRBL framework. Fig. 6C shows that the tool reaches success rates between 89.8% (Golden) and 100% (Urnd) at accuracies between 90.8% (Golden) to 99.4% (Urnd). More detailed values are listed in Supplementary Table S4. The significantly lower performance metrics observed within the Golden dataset can be attributed to the inherent complexity of its reactions, which also include the presence of solvents and catalysts. These elements introduced additional variables into the molecular alignment process, thus posing significant challenges to the predictive capabilities of this framework. In addition, we evaluated the computational efficiency of our methods, observing an average processing time of 46 seconds per 1000 reactions on an average workstation where one-third of the reactions were solved by MCS. In our comparative analysis, our method surpassed the current state-of-the-art, ChemMLM [29], demonstrating superior performance in both success rate and accuracy. The reported outcomes for ChemMLM showed a success rate fluctuating between 4.1 to 42.7% on the USPTO dataset. In contrast, SynRBL demonstrates a remarkable success rate of 99% or higher on the same dataset. Moreover, while the accuracy of ChemMLM varied widely (from 100% for shorter SMILES strings to a mere 8.2% for larger molecules). SynRBL’s accuracy remains robust, largely unaffected by molecular size, and consistently exceeds 98% across the USPTO dataset.

**Fig. 10:**
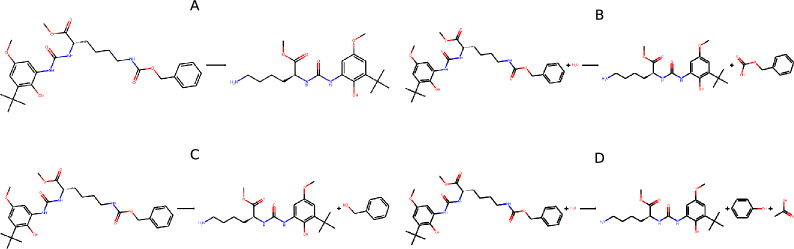
Benchmarking sample. **A** Unbalanced reaction with missing compounds on both sides. **B** Correct solution proposed by SynRBL. **C** Incorrect solution proposed by GPT-4o. **D** Incorrect solution proposed by GPT-4 with stoichiometry balance

The current interest in applications of Large Language Models (LLMs) to problems in chemistry prompted us to explore to what extent such tools could also be employed for reaction rebalancing. We selected a small sample of reactions with a SynRBL solution, illustrated in Fig. 10A and B. We used the same prompt for GPT-3.5, GPT-4o, GPT-4.0, detailed in Table S5. We conducted three trials for each model, which are summarized in Table S6. GPT-3.5 showed the poorest performance, with all three reaction SMILES being invalid. GPT-4o managed to generate valid SMILES, but none of the solutions were balanced (Fig. 10C. GPT-4.0 performed the best, with two of the three solutions correctly adding an additional water molecule on the reactant side, and one solution is balanced but not entirely accurate (Fig. 10D). A common limitation of these models is their method of directly enumerating SMILES, which often fails to accurately represent the molecular topology.

We conclude that although consecutive versions show improvements, LLMs are—at least at present—not capable of reliably rebalancing chemical reactions.

### Application to Reaxys®-derived data

To validate the proposed method in terms of generalizability we tested SynRBL on an extensive dataset derived from Reaxys® 1 [2]. The test dataset consists of 171,913 reactions with at most two products that have balanced Reaxys records. We artificially made these data unbalanced by removing the smaller product molecule in reactions with two products. In addition, all non-carbon compounds are removed from both sides of the reaction. If the same non-carbon compound is present multiple times, it is only removed once in order not to lose essential compounds on both sides of the reaction. These artificially unbalanced reactions were then rebalanced with SynRBL and compared to the initial reaction. This is a conservative evaluation because only exact (canonicalized) matches count as correct. An unbalanced reaction might have multiple equally viable solutions. However, checking if a structurally different solution is also chemically correct is non-trivial.

Table 4 contains a summary of the performance on the Reaxys test set. For MCS-based method and the overall result, the table contains one column without the confidence prediction and one for the prediction with a confidence threshold of 50%. The largest difference in performance in comparison with the datasets discussed above was observed for the MCS-based method. The Rule-based method achives comparable success rates and accuracies. One reason for the lower success rate of the MCS-based method on Reaxys data compared to the other datasets is a different charge representation in some reaction SMILES. A higher diversity of reaction types and more complex structures probably also contributes to a higher rate of failure. On the other hand, our procedure to “unbalance” the reactions is likely to be unrealistically brutal and may have deleted salient information that would have been retained in manually produced reaction data records. We suspect that these performance data hence are in fact lower bounds.

**Table 4:** Reaxys performance with and without confidence prediction

	Rule-based	MCS-based	MCS-based	SynRBL	SynRBL
		$\text {conf.} \ge 0$%	$\text {conf.} \ge 50$%	$\text {conf.}\ge 0$%	$\text {conf.} \ge 50$%
Input Reactions	83366	88595	88595	171913	171913
Solved Reactions	78692	51828	35491	130520	114183
Correct Reactions	77088	27439	22007	104527	99095
Success Rate	94.39%	58.50%	40.06%	75.92%	66.42%
Accuracy	97.96%	52.94%	62.01%	80.09%	86.79%

## Conclusion

In this contribution, we investigated the SynRBL framework as an innovative approach for the rebalance of incomplete reaction entries in chemical databases. SynRBL combines a rule-based approach for carbon-balanced reactions and the MCS-based workflow for carbon-unbalanced reactions. The latter combines variants of the MCIS and MCES problem to increase the fraction of instances in which chemically correct subgraph embedding is found. For the MCS-based component, moreover, a trained feature-based machine learning model was used to estimate the prediction confidence. SynRBL was rigorously evaluated based on five meticulously curated validation datasets, encompassing a subset of the Golden dataset, the Jaworski dataset, and three variants of the USPTO 50k database. Overall, the framework achieves unprecedented accuracy, exceeding 99% on the subset of database entries that it can process successfully. These cover more than 90% of the unbalanced reactions in the datasets used for evaluation. As a by-product of the rule-based analysis, we observed that the signature *O* : 1, *Q* : 0 referring to a single oxygen is a strong indication for an error in the database entry.

The current implementation of SynRBL is limited to product-dominated or reactant-dominant reaction entries. Moreover, it does not cover certain types of carbon-unbalanced reactions, in particular multi-step reactions, cyclizations and other complex rearrangement reactions that are difficult for the MCS-based branch of the framework. The SynRBL software is designed, however, to facilitate future extensions of the rule sets as well as of the MCS strategies. SynRBL is not based on a machine learning approach. Instead, it makes use of “textbook-level” knowledge of chemical reactions in combination with conceptually simple optimization problems. While it does not cover all situations and hence leaves a few percent of the database entries unbalanced, this approach has the advantage of being independent of specific training data and thus of biases inherent in specific data sources. We observed that it indeed yields robust results for datasets with very different chemical content.

A practical issue in processing reaction data is that multiple *alternative* products, typically isomers, may be included in a single data record. Such data need to be expanded into separate reaction schemes before rebalancing. Since there is no standardized data format for such cases, customized preprocessing specific to the data source is required before SynRBL can be invoked. It remains an interesting open question whether multiple products from the same reaction can provide additional information that is helpful for correct rebalancing. On the one hand, one could introduce a generalized sorting function that ranks solutions based on predictions for the alternative products. However, it cannot be assured that all alternative reactions will yield identical by-products, thereby rendering this approach potentially ineffective and necessitating varied rebalancing strategies.

Reaction rebalancing with SynRBL can provide much larger and more diverse sets of stoichiometrically balanced reactions as a basis for a wide variety of data-driven tasks in cheminformatics. In particular, we expect that better atom-atom maps can be obtained from such balanced data since the mappers are freed from the need to solve the reaction balancing problem simultaneously. We expect beneficial effects also on learning approaches, e.g. in forward prediction, retrosynthesis planning, and, notably, the elucidation of reaction mechanisms. Finally, representations of reaction mechanisms in the form of graph transformation rules [52] could be employed as an orthogonal validation strategy, particularly on data sources where *named reactions* are annotated in the metadata.

### Supplementary information

Supplementary Material 1.

Supplementary Material 2.

## Data Availability

The datasets supporting the conclusions of this article are available in the SynRBL repository: https://github.com/TieuLongPhan/SynRBL/tree/main/Data. The source code is avaiable at: https://github.com/TieuLongPhan/SynRBL.

## Footnotes

1 Copyright ©2022 Elsevier Limited except certain content provided by third parties. Reaxys is a trademark of Elsevier Limited.
