# Supplementary material for: Reaction rebalancing: a novel approach to curating reaction databases
Source: J Cheminform. 2024 Jul 19;16:82. doi: 10.1186/s13321-024-00875-4 (PMC11264917; doi:10.1186/s13321-024-00875-4)
Supplement: Supplementary file 1 — Supplementary Material 1. [file 13321_2024_875_MOESM1_ESM.pdf]

Supplementary Information

Method Details

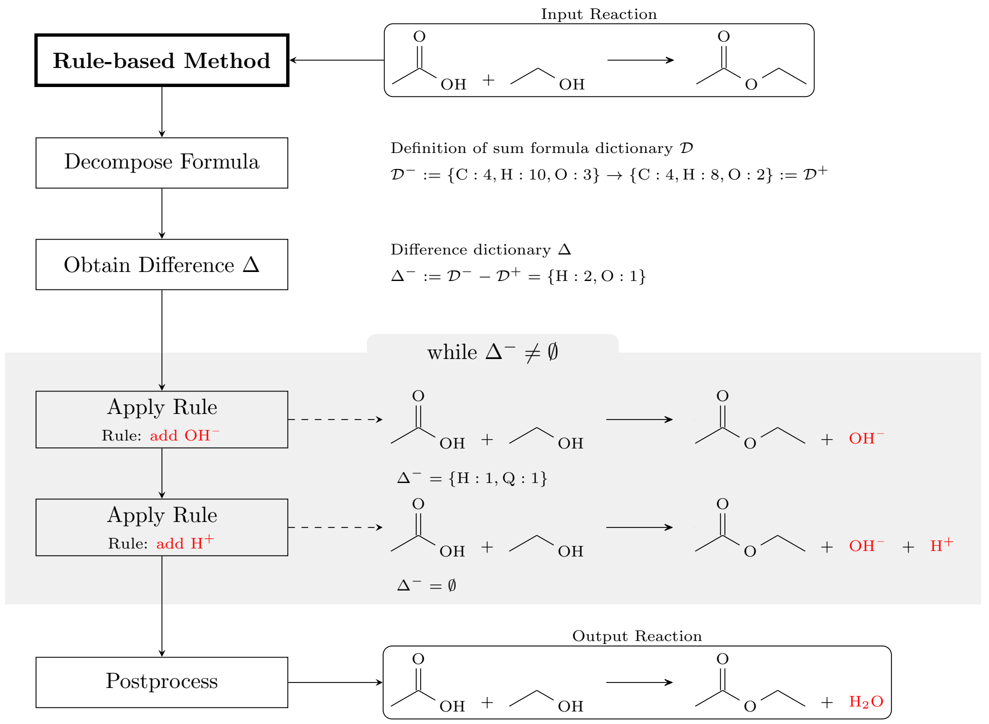

Fig. S1: Flowchart describing the functional steps of the Rule-based method. The first step is decomposing the structured data into an atom-count dictionary for both sides of the reaction equation. These representations are used to obtain the difference Δ. Subsequently, rules are applied until the reactants and products are the same.

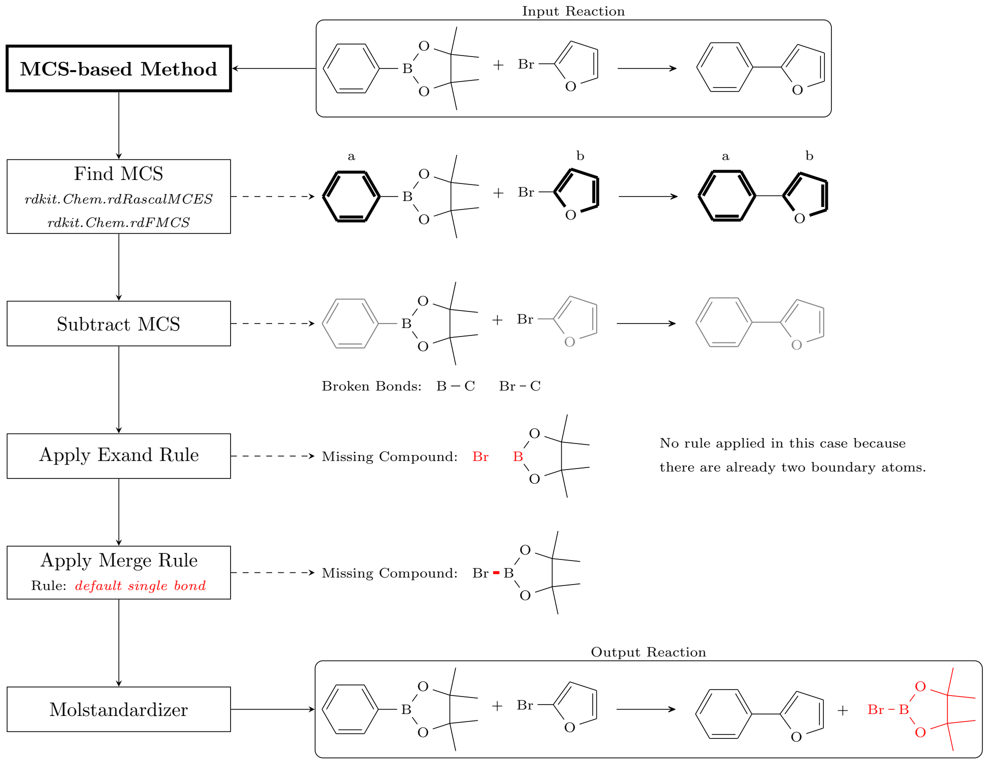

Fig. S2: Flowchart describing the functional steps of the MCS-based method. The first step is finding the maximum common subgraph between reactants and product. This step utilizes algorithms from `RDKit` depending on the specified matching configuration. The next section and Fig. S3 describe why multiple configurations are used. The MCS result is subtracted from the input to get the missing structure. This leaves a set of fragments with open boundaries, i.e. the broken bonds. To obtain a chemically reasonable result a set of expand and merge rules are applied. In the shown example no expand rule is needed because there are already two boundaries. A merge rule creates a single bond between the two fragments yielding the correct missing compound that can then be added to the reactants.

Comparison of the MCS Variants

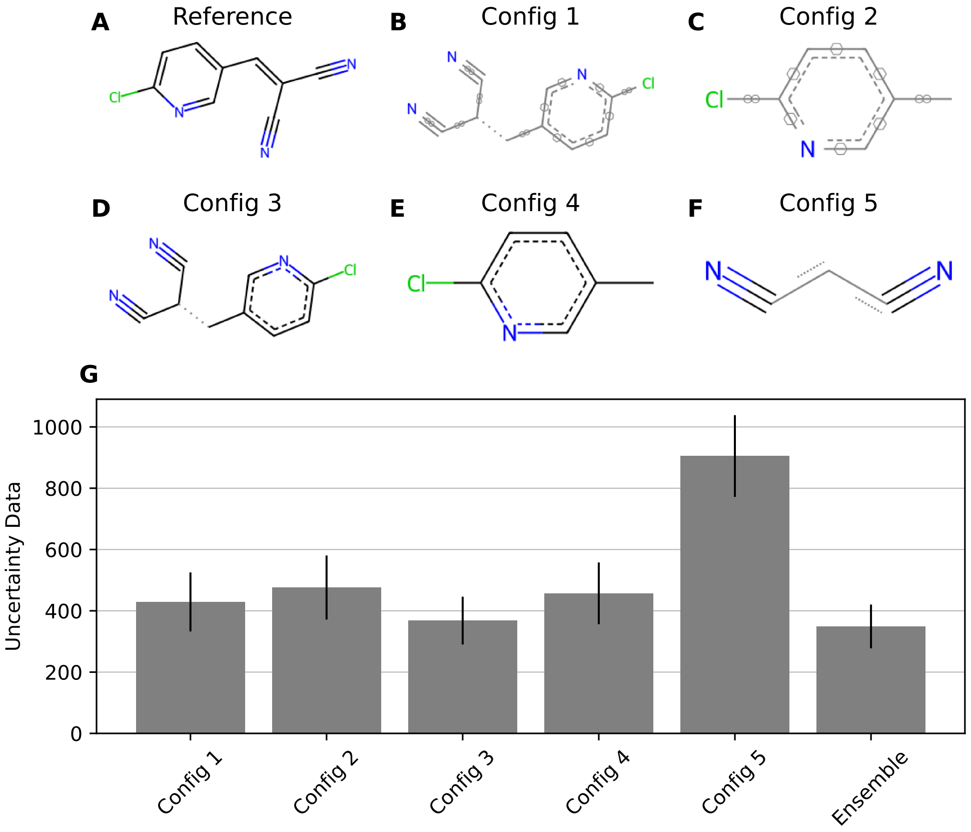

Fig. S3: Benchmarking analysis of MCS search configuration. (A) represents the reference molecules. (B-F) illustrate the MCS results from various configurations. (G) demonstrates the comparative analysis among different configurations and an ensemble method.

As described in the main text, the MCS problem was solved in several different versions ("configurations"), none of which is guaranteed to always identify the chemically correct common subgraph. We benchmarked the different variants and found that they are at least in part complementary. As depicted in Fig. S3, spanning panels A through F, three distinct cases of MCS were identified, where configurations 1 to 4 were MCIS, while configuration 5 was MCES. Notably, the MCES approach demonstrated a capability to expedite the resolution of the NP-hard subgraph isomorphism problem more efficiently than its MCIS counterpart. However, its performance efficacy was suboptimal, a trend observable in Fig. S3G. This discrepancy is likely due to the significant role of bond modifications in chemical reactions, highlighting the

dependence of the MCES search on bond-defined substructures. Remarkably, Configuration 3 achieved superior performance, disregarding bond order and complete rings, excluding comparisons with ensemble methods.

These finds emphasize the well-known fact that any particular variant of the graph-theoretical MCS problem does not always identify the chemically correct atom correspondences between molecular graphs. The combination of multiple variations, as implemented in the ensemble method, can achieve at least a moderate improvement, Figure S3G. However, given the additional computational cost of computing multiple MCS solutions, Configuration 3 appears to be the best pragmatic choice given its performance and reduced computational requirements. This observation that the ensemble approach improved chemical correctness, albeit slightly, however, can serve as a natural starting point for the development of an improved combinatorial atom-atom-mapping method.

Additional Figures and Tables

Table S1: Merge Rules; FG: Functional Group

Cond. u	Cond. v	Action u	Action v	Bond
**O**<br>FG: Carbonyl	**P**<br>Pattern: P=O	-	change_bond<br>P=O to P-O	double
**O**<br>FG: Carbonyl	**P**<br>Pattern: !P=O	-	-	double
**O**<br>FG: Enol, Alcohol, Phenol	**P**	-	-	single
**S**	**X**	-	-	no bond
**N,O,X**	**N,O,X**	-	-	no bond
*	*	-	-	single

Table S2: Expand Rules; FG: Functional Group; cut edge: u - v

Cond. u	Cond. v	FG	Expand
C	O	Ether	I
C	S	Thioether	I
C	O	Ester	O
C	S	Thioester	O
C	N	Amide	O
Mg, Zn, Si, B	*	*	O
O	!O, !N	*	O
N	!O, !N	*	O
C	C	*	O

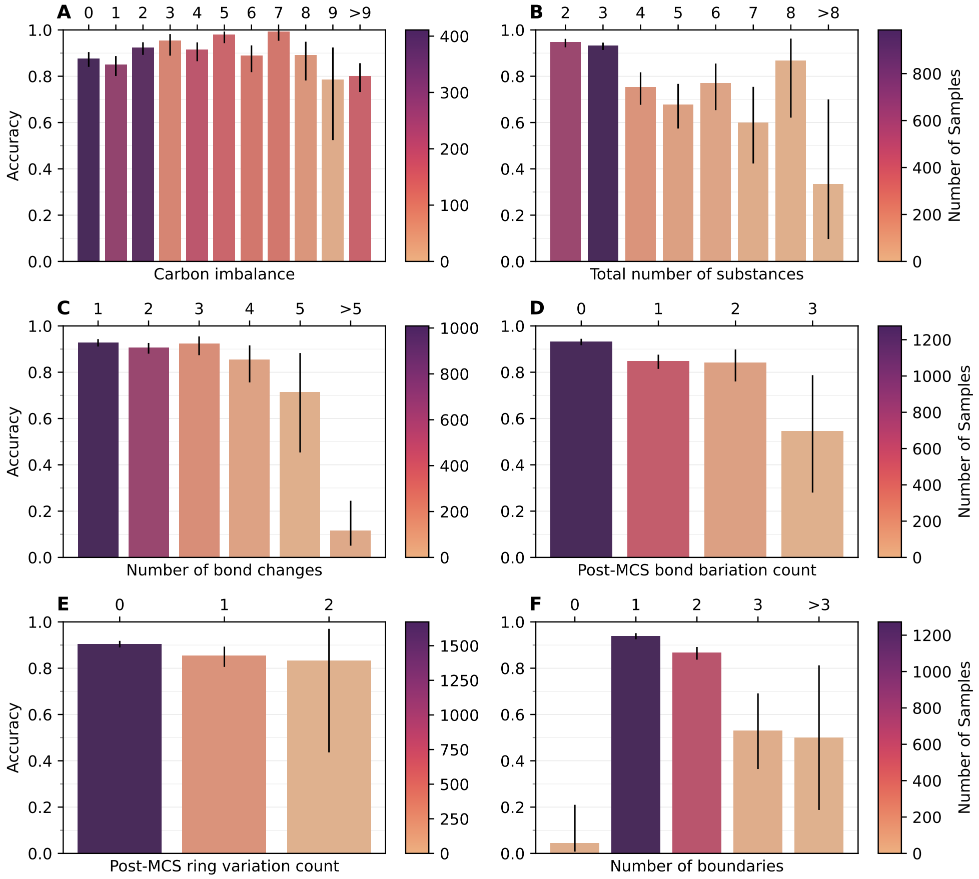

Fig. S4: Exploratory data analysis of MCS-based method performance. (A) Accuracy fluctuates slightly and declines when carbon imbalance exceeds seven. (B) The method performs best with less than four substances. (C) Accuracy drops with over five bond changes, indicating difficulty with rearrangement reactions. (D) Post-MCS bond differences between reactants and products show a decreasing trend similar to bond changes, with optimal performance below three. (E) Ring differences between reactants and products post-MCS show a minor decreasing trend with an increasing number of ring differences. (F) The detection of boundary atoms or reaction centers by MCS is crucial; the method fails without boundary atom detection and underperforms when the number exceeds two.

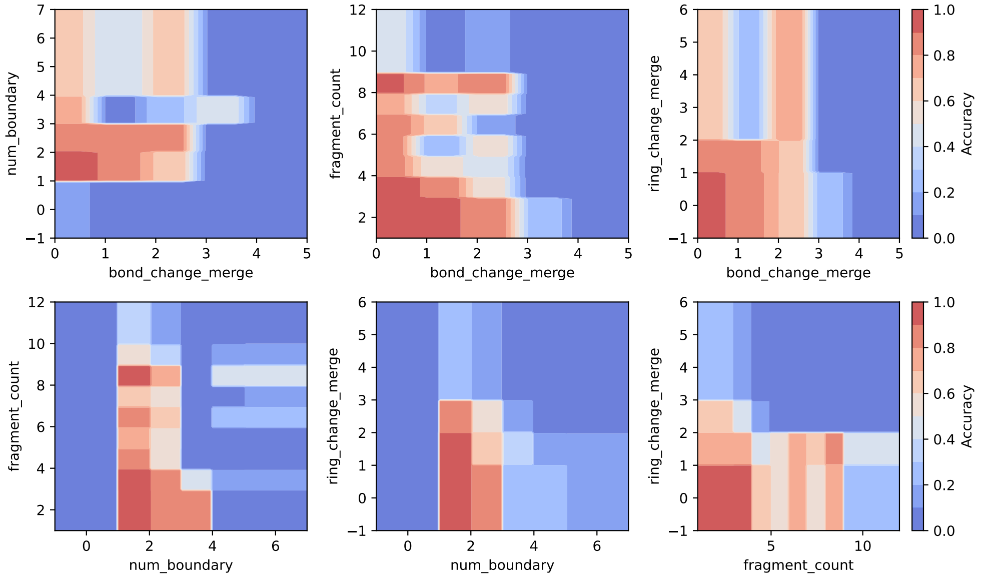

Fig. S5: Contour plots illustrate the confidence region formed by pairs of features. The warm colors in the contour plot represent regions of high confidence, indicating areas where our method demonstrates high accuracy. Conversely, the cool colors denote regions of lower confidence, reflecting areas where our method's accuracy is comparatively lower.

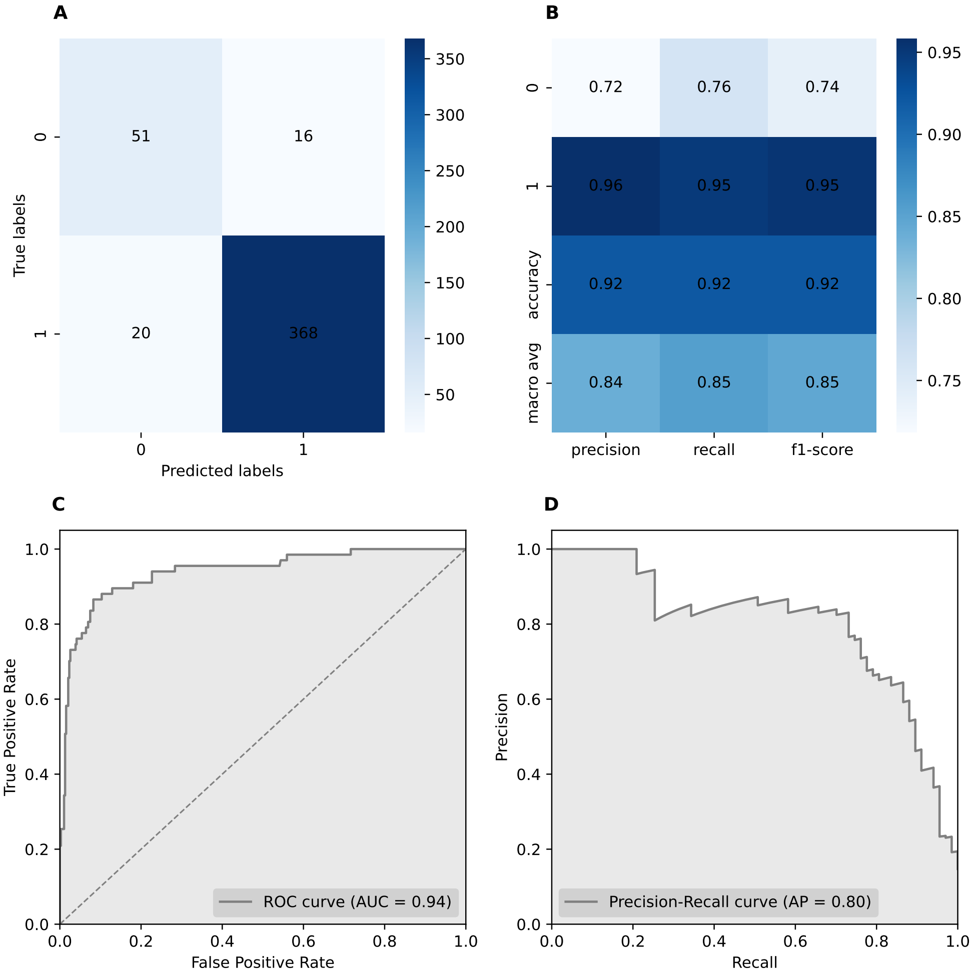

Fig. S6: Evaluation of model performance for a confidence level model using `XGBoost` and `SMOTETomek`. (A) The confusion matrix shows the number of actual versus predicted values. (B) The classification report provides performance metrics, including an F1 score of 0.91. (C) The ROC curve is presented with an AUC of 0.94. (D) The precision-recall curve is shown, with an average precision of 0.8.

Table S3: Library of substitution rules $\hat{r} \rightsquigarrow X_r$ for Section 2.2.2

X_r Formula	SMILES	$\hat{r}$ Composition
O	[O]	{O: 1, Q: 0}
Cl_2	ClCl	{Cl: 2, Q: 0}
N_3^-	[N-]=[N+]=[N-]	{N: 3, Q: -1}
H	[H]	{H: 1, Q: 0}
F_2	FF	{F: 2, Q: 0}
Cl_2	ClCl	{Cl: 2, Q: 0}
Br_2	BrBr	{Br: 2, Q: 0}
I_2	II	{I: 2, Q: 0}
H^+	[H+]	{H: 1, Q: 1}
Na^+	[Na+]	{Na: 1, Q: 1}
Li^+	[Li+]	{Li: 1, Q: 1}
K^+	[K+]	{K: 1, Q: 1}
Ca^{2+}	[Ca+2]	{Ca: 1, Q: 2}
Mg^{2+}	[Mg+2]	{Mg: 1, Q: 2}
Ba^{2+}	[Ba+2]	{Ba: 1, Q: 2}
Al^{3+}	[Al+3]	{Al: 1, Q: 3}
Zn^{2+}	[Zn+2]	{Zn: 1, Q: 2}
Cu^{2+}	[Cu+2]	{Cu: 1, Q: 2}
Cu^+	[Cu+]	{Cu: 1, Q: 1}
F^-	[F-]	{F: 1, Q: -1}
Cl^-	[Cl-]	{Cl: 1, Q: -1}
Br^-	[Br-]	{Br: 1, Q: -1}
I^-	[I-]	{I: 1, Q: -1}
N_2	N#N	{N: 2, Q: 0}
O_2	O=O	{O: 2, Q: 0}
S^{2-}	[S-2]	{S: 1, Q: -2}
H_3N	N	{N: 1, H: 3, Q: 0}
H_2O	O	{O: 1, H: 2, Q: 0}
H_2O_2	OO	{O: 2, H: 2, Q: 0}
H_4N^+	[NH4+]	{N: 1, H: 4, Q: 1}
OH^-	[OH-]	{O: 1, H: 1, Q: -1}
NH_3	N	{N: 1, H: 3, Q: 0}
NO_2^-	O=N[O-]	{N: 1, O: 2, Q: -1}
NO_3^-	[N+](=O)([O-])[O-]	{N: 1, O: 3, Q: -1}
NH_2^-	[NH2-]	{N: 1, H: 2, Q: -1}
SO_4^{2-}	[O-]S(=O)(=O)[O-]	{S: 1, O: 4, Q: -2}
PO_4^{3-}	[O-]P(=O)([O-])[O-]	{P: 1, O: 4, Q: -3}
SO_3^{2-}	[O-]S(=O)[O-]	{S: 1, O: 3, Q: -2}
IO_3^-	[O-]I(=O)=O	{I: 1, O: 3, Q: -1}
H_3NO	NO	{N: 1, O: 1, H: 3, Q: 0}
H_4NO^+	[NH3+]O	{N: 1, O: 1, H: 4, Q: 1}
$B(OH)_3$	B(O)(O)O	{B: 1, O: 3, H: 3, Q: 0}
H_3BO_2	B(O)(O)	{B: 1, O: 2, H: 3, Q: 0}
CO_2	C=O	{C: 1, O: 2, Q: 0}
$SOCl_2$	O=S(Cl)Cl	{S: 1, O: 1, Cl: 2, Q: 0}
$H_4N_2O_2S$	NS(N)(=O)=O	{N: 2, S: 1, O: 2, H: 4, Q: 0}
$HClO_3S$	O=S(=O)(O)Cl	{S: 1, O: 3, Cl: 1, H: 1, Q: 0}
$B(OH)_2Cl$	B(O)(O)Cl	{B: 1, O: 2, H: 2, Cl: 1, Q: 0}
$B(OH)_2Br$	B(O)(O)Br	{B: 1, O: 2, H: 2, Br: 1, Q: 0}
$B(OH)_2I$	B(O)(O)I	{B: 1, O: 2, H: 2, I: 1, Q: 0}
H_2ClNO_2S	NS(=O)(=O)Cl	{N: 1, S: 1, O: 2, Cl: 1, H: 2, Q: 0}

Table S4: Comprehensive Performance Metrics of `SynRBL`

**Dataset**	**Jaworski**	`Golden`	`Uunb`	`Urnd`	`Udiff`
Total number reactions	637	1851	540	803	1589
Number of unbalance reactions	335	1642	540	803	1589
Number of rule solved reactions	181	754	240	324	1134
Rule success rate (%)	89.6	93.55	97.96	99.69	96.1
Number of rule accurate reactions	179	752	239	322	1133
Rule accuracy (%)	98.9	99.73	99.58	99.38	99.91
Number of MCS solved reactions	127	721	298	479	451
MCS success rate (%)	82.47	81.19	99.33	100	99.12
Number of MCS accurate reactions	121	588	289	476	437
MCS accuracy (%)	95.28	81.55	96.98	99.37	96.9
All solved reactions	308	1475	538	803	1585
All success rate (%)	91.94	89.83	99.63	100	99.75
All accurate reactions	300	1340	528	798	1570
All accuracy (%)	97.40	90.85	98.14	99.38	99.05

Table S5: ChatGPT prompt for chemical rebalancing task

"As a computational chemist, you're tasked with a challenge involving a SMILES representation of a chemical reaction that is currently unbalanced. Please analyze the provided reaction SMILES and identify any missing compounds. Your goal is to modify and balance the reaction by adding the appropriate compounds. Return the corrected, balanced reaction SMILES."

Initial Reaction SMILES:

`COC(=O)[C@H](CCCCNC(=O)OCc1ccccc1)NC(=O)Nc1cc(OC)cc(C(C)(C)C)c1O >>`
`COC(=O)[C@H](CCCCN)NC(ofO)Nc1cc(OC)cc(C(C)(C)C)c1O.`

"Please provide the new, balanced reaction SMILES after your adjustments."

Table S6: LLM Benchmarking

**Description**	**Reaction SMILES**
Initial Reaction	COC(=O)[C@H](CCCCNC(=O)OCc1ccccc1)NC(=O)Nc1cc(OC)cc(C(C)(C)C)c1O>><br>COC(=O)[C@H](CCCCN)NC(=O)Nc1cc(OC)cc(C(C)(C)C)c1O
SynRBL Solution	COC(=O)[C@H](CCCCNC(=O)OCc1ccccc1)NC(=O)Nc1cc(OC)cc(C(C)(C)C)c1O.O>><br>COC(=O)[C@H](CCCCN)NC(=O)Nc1cc(OC)cc(C(C)(C)C)c1O.O=C(O)OCc1ccccc1
GPT-3.5 Solution 1	COC(=O)C@HNC(=O)Nc1cc(OC)cc(C(C)(C)C)c1O >><br>COC(=O)C@HNC(=O)Nc1cc(OC)cc(C(C)(C)C)c1O.CCCCNC
GPT-3.5 Solution 2	COC(=O)C@HNC(=O)Nc1cc(OC)cc(C(C)(C)C)c1O + H2O>><br>COC(=O)C@HNC(=O)Nc1cc(OC)cc(C(C)(C)C)c1O + NH2C(=O)OH
GPT-3.5 Solution 3	COC(=O)C@HNC(=O)Nc1cc(OC)cc(C(C)(C)C)c1O >><br>COC(=O)C@HNC(=O)Nc1cc(OC)cc(C(C)(C)C)c1O + CCCCNC(=O)OCc1ccccc1
GPT-4o Solution 1	COC(=O)[C@H](CCCCNC(=O)OCc1ccccc1)NC(=O)Nc1cc(OC)cc(C(C)(C)C)c1O>><br>COC(=O)[C@H](CCCCN)NC(=O)Nc1cc(OC)cc(C(C)(C)C)c1O.OCc1ccccc1
GPT-4o Solution 2	COC(=O)[C@H](CCCCNC(=O)OCc1ccccc1)NC(=O)Nc1cc(OC)cc(C(C)(C)C)c1O>><br>COC(=O)[C@H](CCCCN)NC(=O)Nc1cc(OC)cc(C(C)(C)C)c1O.c1ccccc1C(=O)O.CO
GPT-4o Solution 3	CCOC(=O)[C@H](CCCCNC(=O)OCc1ccccc1)NC(=O)Nc1cc(OC)cc(C(C)(C)C)c1O>><br>COC(=O)[C@H](CCCCN)NC(=O)Nc1cc(OC)cc(C(C)(C)C)c1O.OCc1ccccc1
GPT-4.0 Solution 1	COC(=O)[C@H](CCCCNC(=O)OCc1ccccc1)NC(=O)Nc1cc(OC)cc(C(C)(C)C)c1O.O>><br>COC(=I)[C@H](CCCCN)NC(=O)Nc1cc(OC)cc(C(C)(C)C)c1O.OCc1ccccc1.<br>CO.O=C=O
GPT-4.0 Solution 2	COC(=O)[C@H](CCCCNC(=O)OCc1ccccc1)NC(=O)Nc1cc(OC)cc(C(C)(C)C)c1O>><br>COC(=O)[C@H](CCCCN)NC(=O)Nc1cc(OC)cc(C(C)(C)C)c1O.O=C(O)Cc1ccccc1
GPT-4.0 Solution 3	COC(=O)[C@H](CCCCNC(=O)OCc1ccccc1)NC(=O)Nc1cc(OC)cc(C(C)(C)C)c1O.O>><br>COC(=O)[C@H](CCCCN)NC(=O)Nc1cc(OC)cc(C(C)(C)C)c1O.Oc1ccccc1.CC(=O)O
