# Supplementary material for: Reaction rebalancing: a novel approach to curating reaction databases
Source: J Cheminform. 2024 Jul 19;16:82. doi: 10.1186/s13321-024-00875-4 (PMC11264917; doi:10.1186/s13321-024-00875-4)

Original Reaction golden_dataset_38

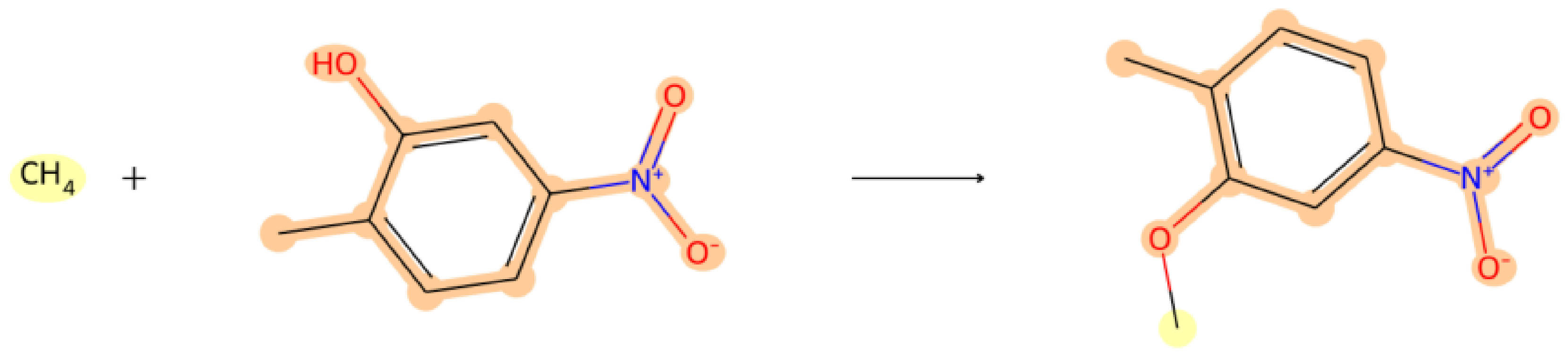

Imputed Reaction golden_dataset_38

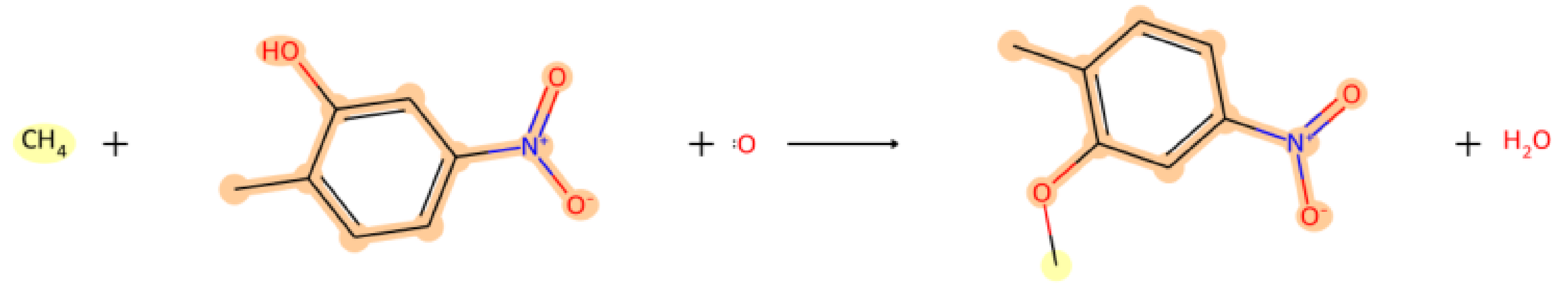

Ground Truth golden_dataset_38

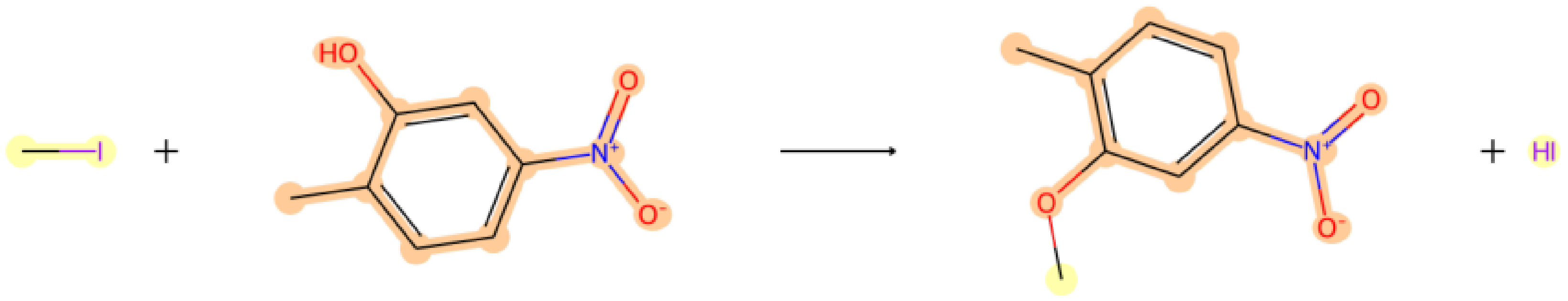

Original Reaction golden_dataset_460

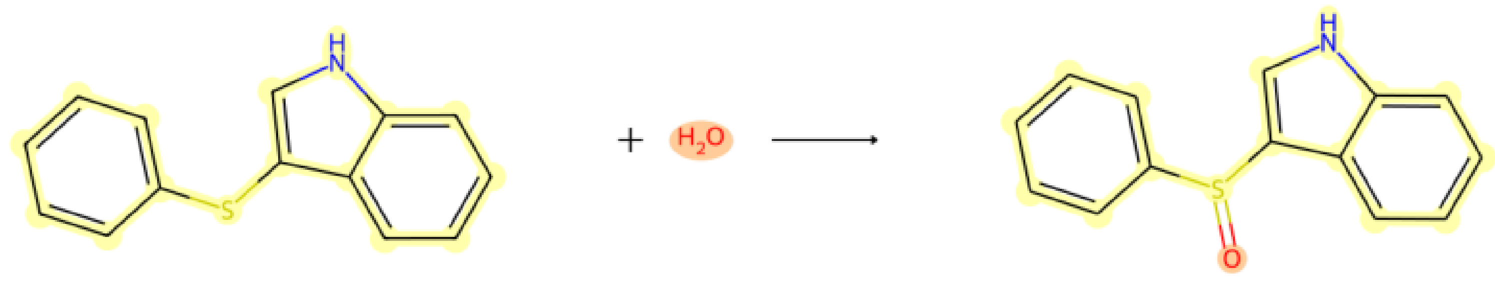

Imputed Reaction golden_dataset_460

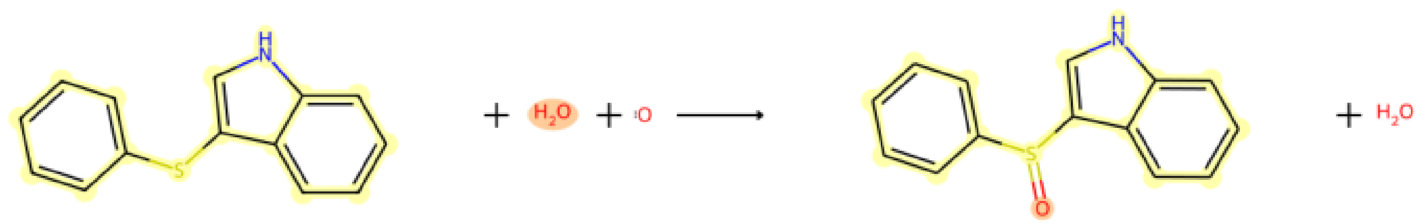

Ground Truth golden_dataset_460

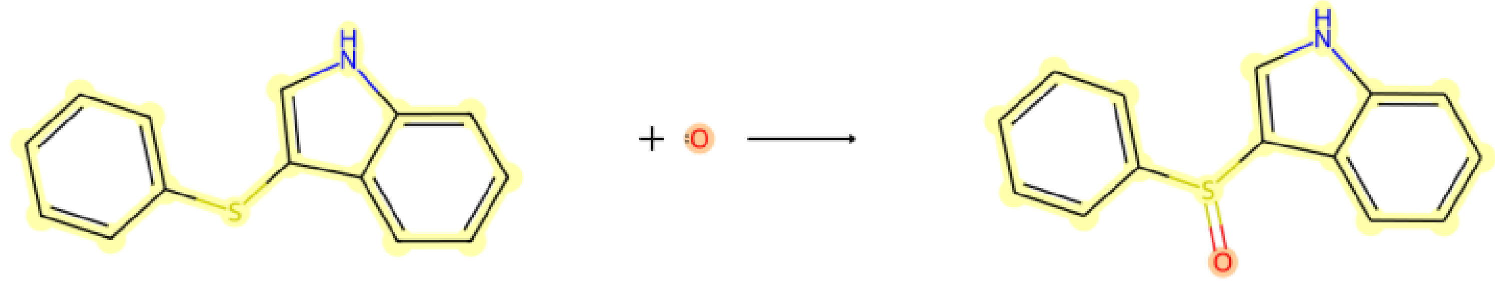

Original Reaction golden_dataset_429

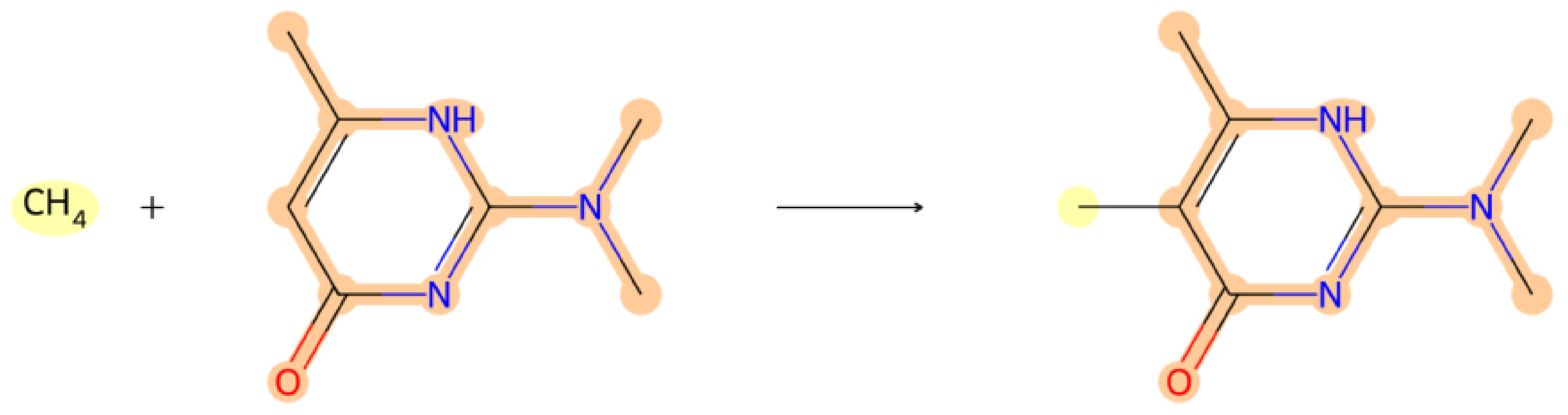

Imputed Reaction golden_dataset_429

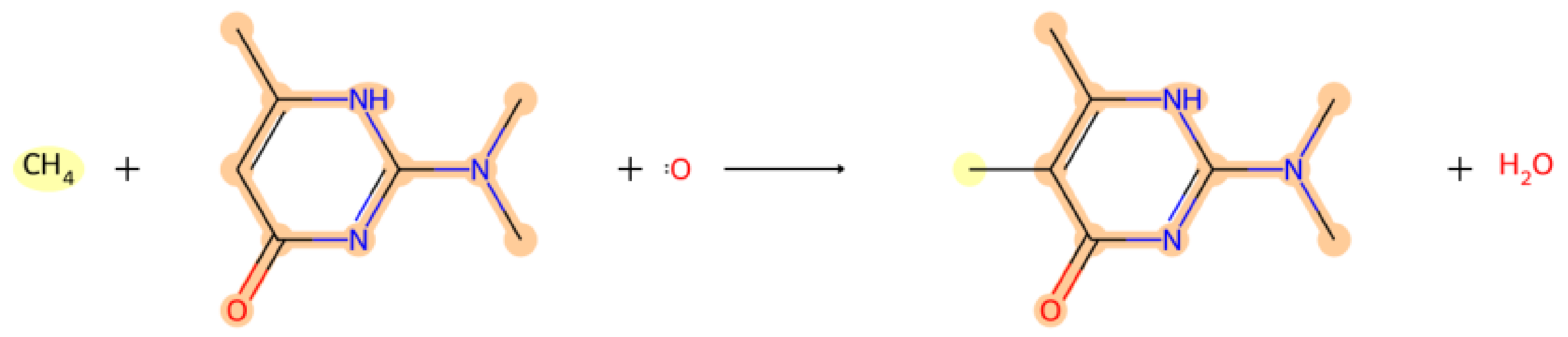

Ground Truth golden_dataset_429

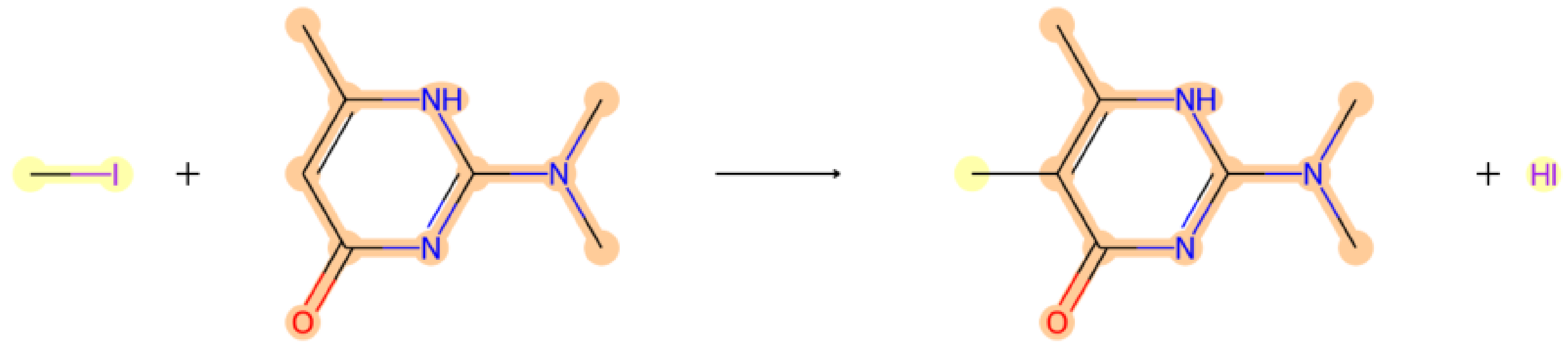

Original Reaction golden_dataset_441

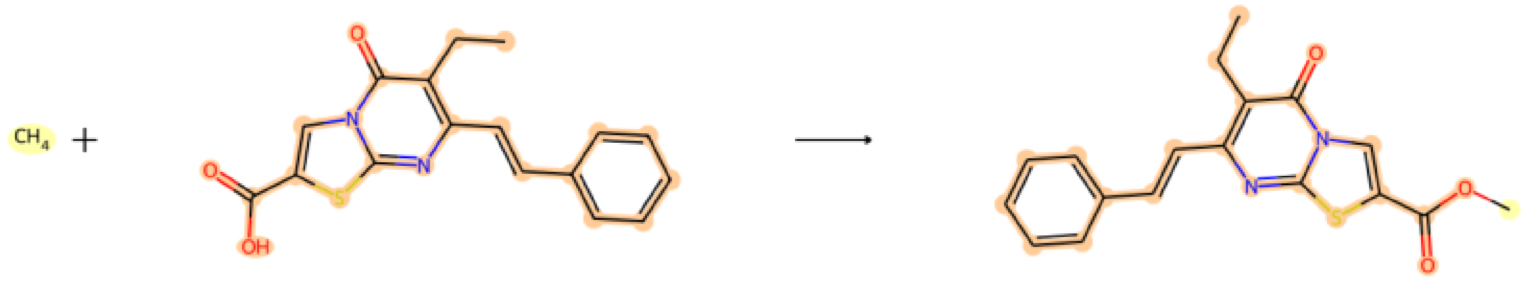

Imputed Reaction golden_dataset_441

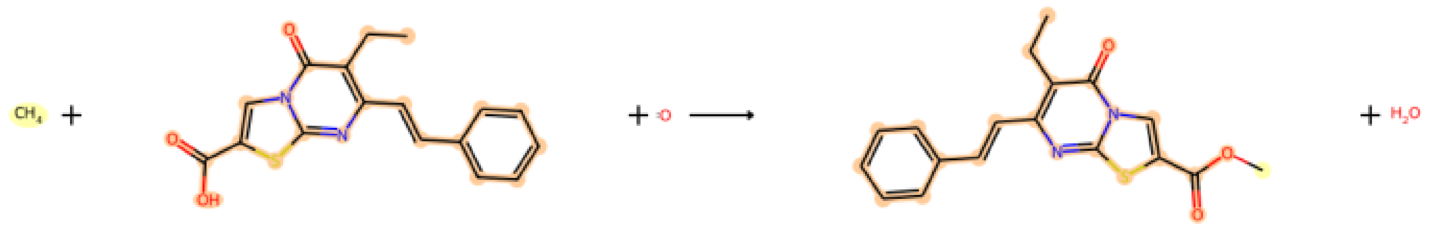

Ground Truth golden_dataset_441

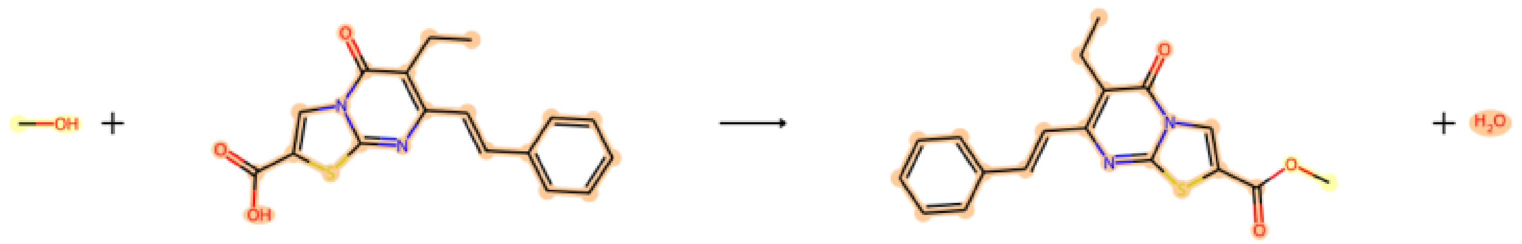

Original Reaction golden_dataset_445

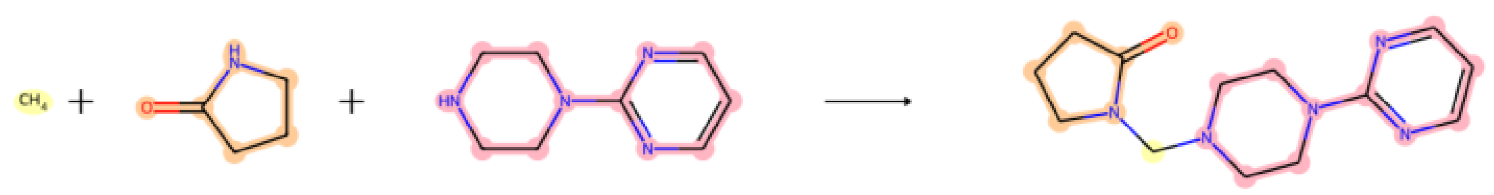

Imputed Reaction golden_dataset_445

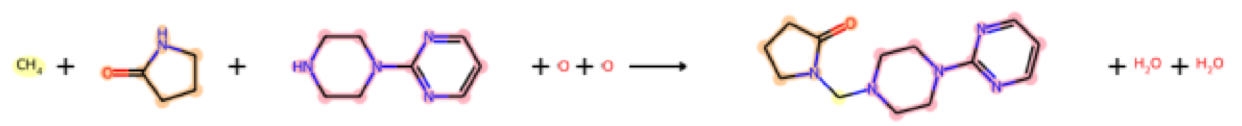

Ground Truth golden_dataset_445

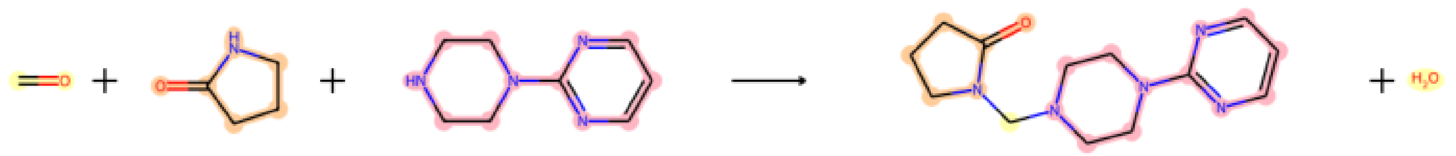

Original Reaction golden_dataset_450

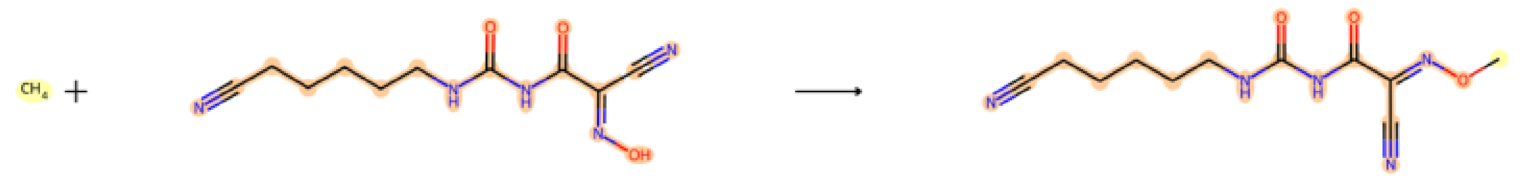

Imputed Reaction golden_dataset_450

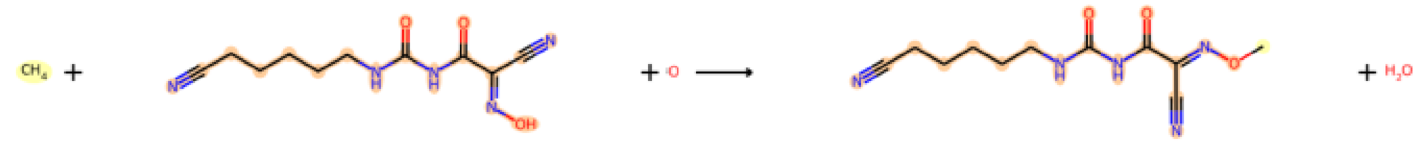

Ground Truth golden_dataset_450

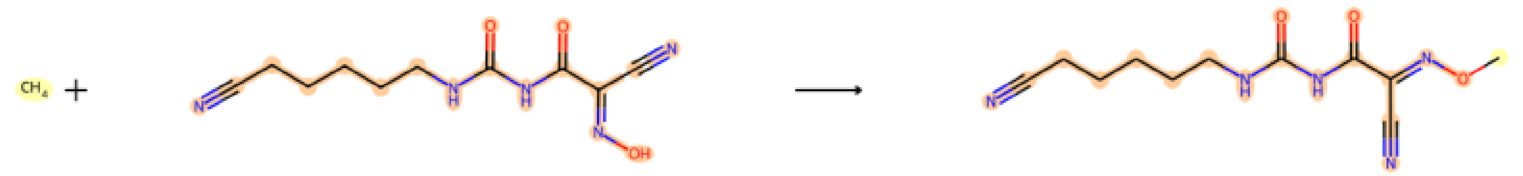

Original Reaction golden_dataset_459

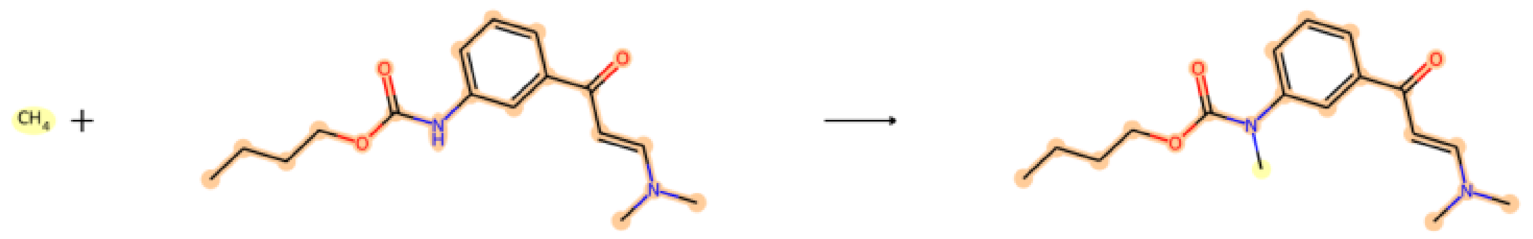

Imputed Reaction golden_dataset_459

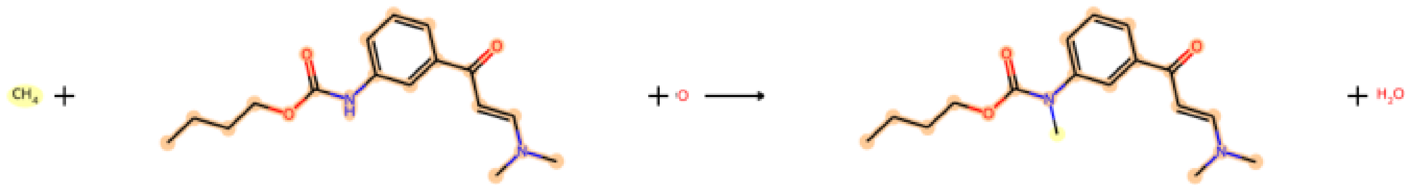

Ground Truth golden_dataset_459

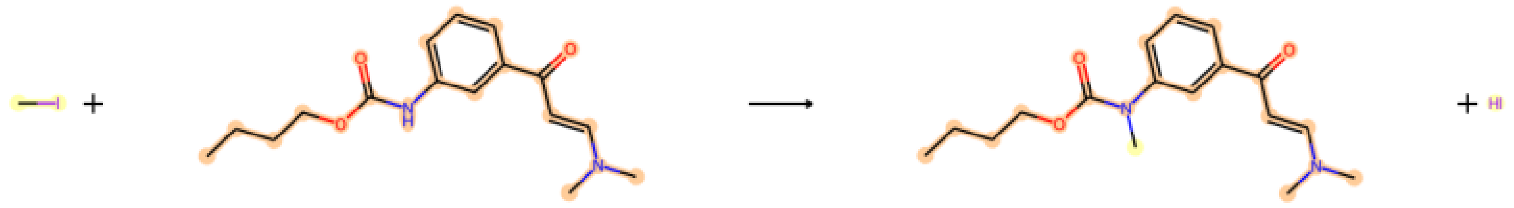

Original Reaction golden_dataset_465

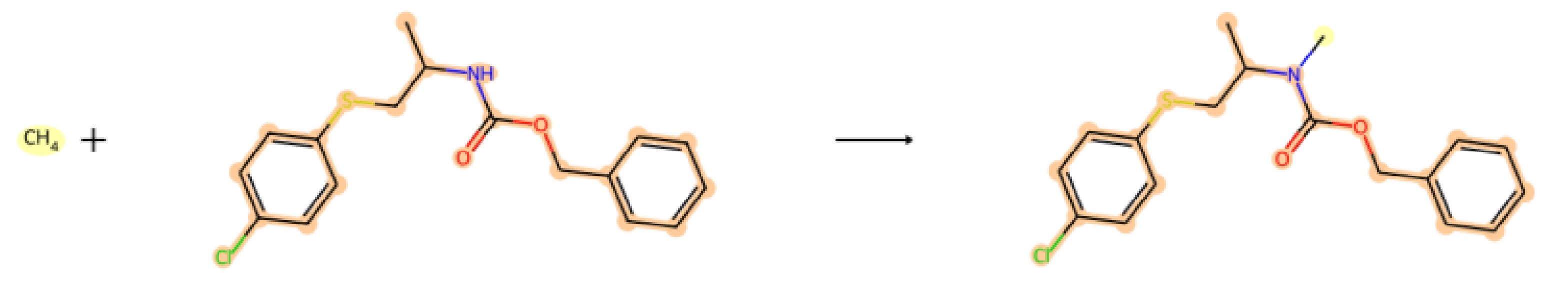

Imputed Reaction golden_dataset_465

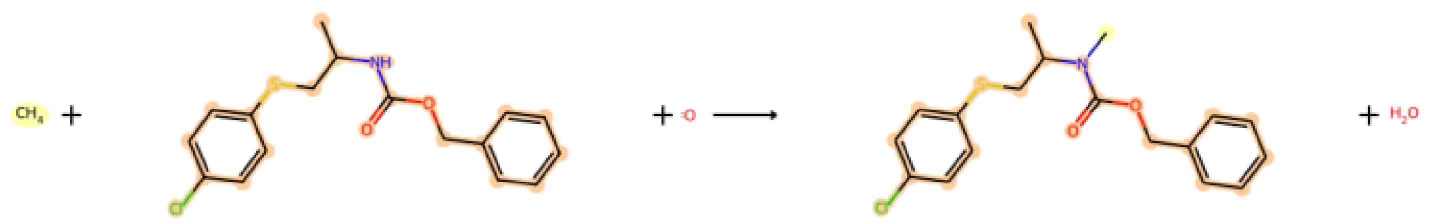

Ground Truth golden_dataset_465

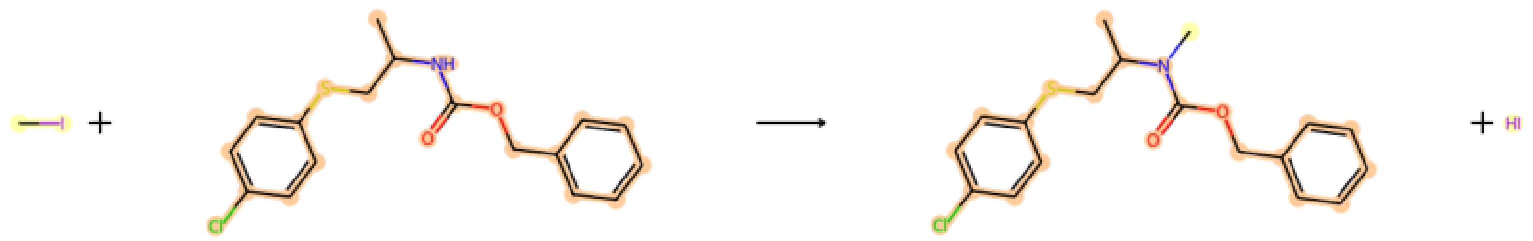

Original Reaction golden_dataset_466

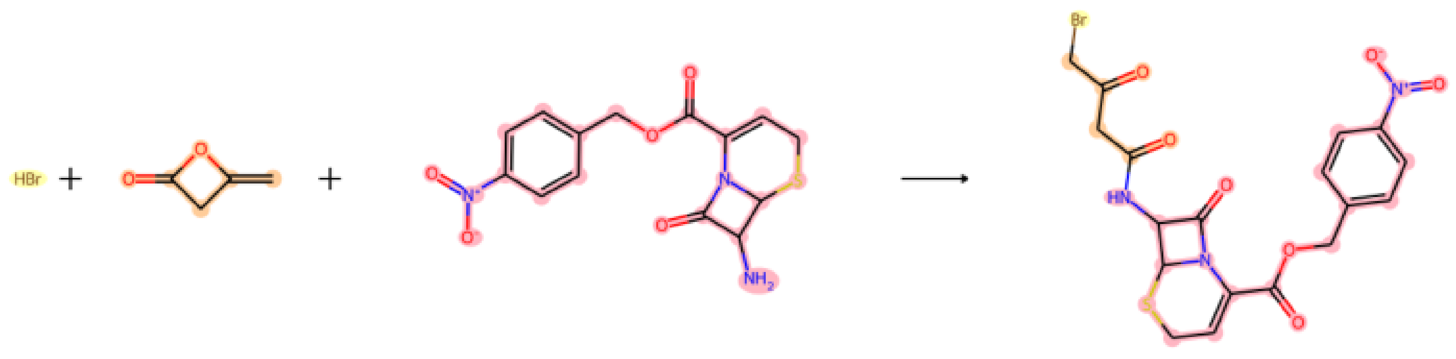

Imputed Reaction golden_dataset_466

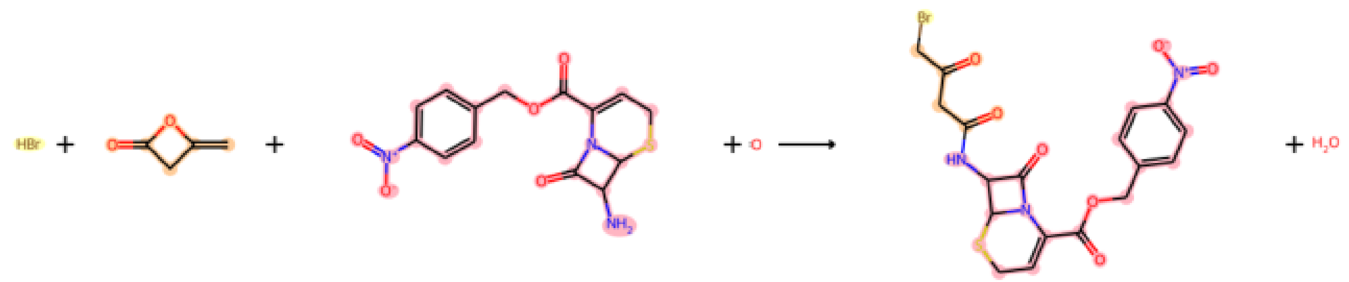

Ground Truth golden_dataset_466

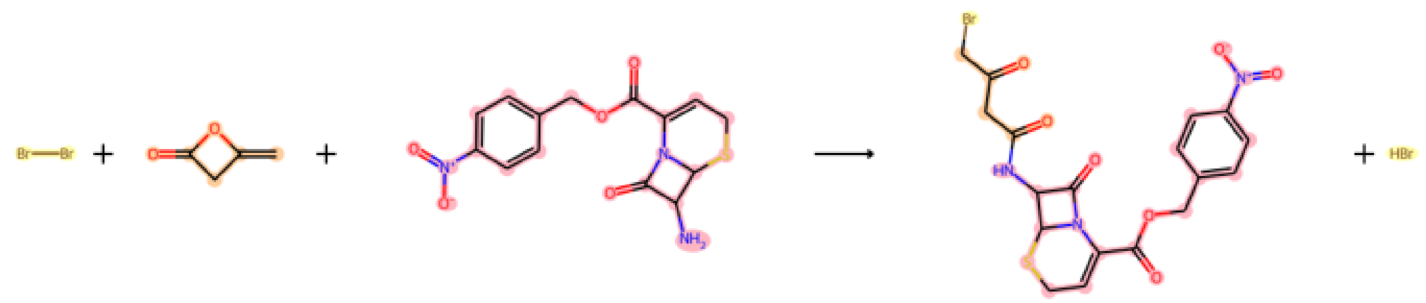

Original Reaction golden_dataset_467

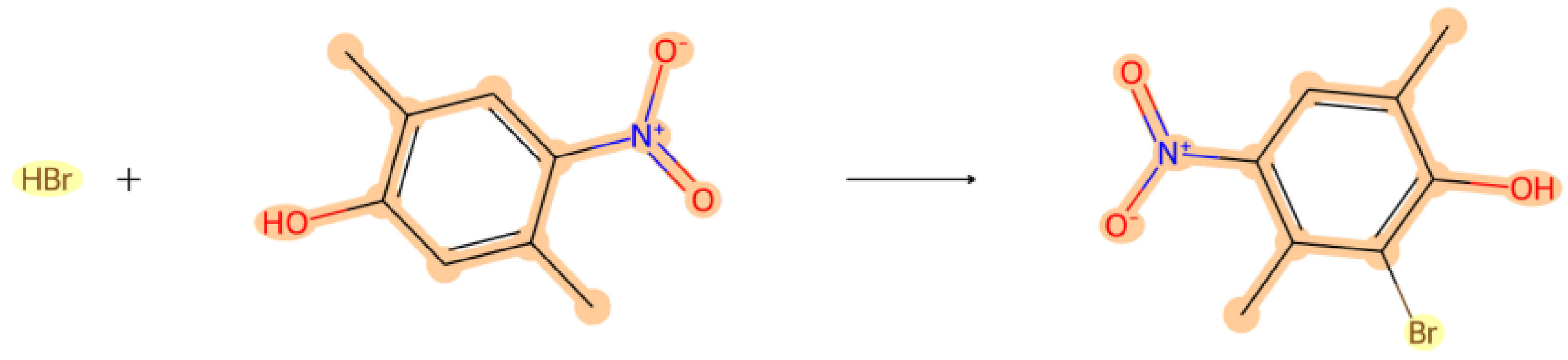

Imputed Reaction golden_dataset_467

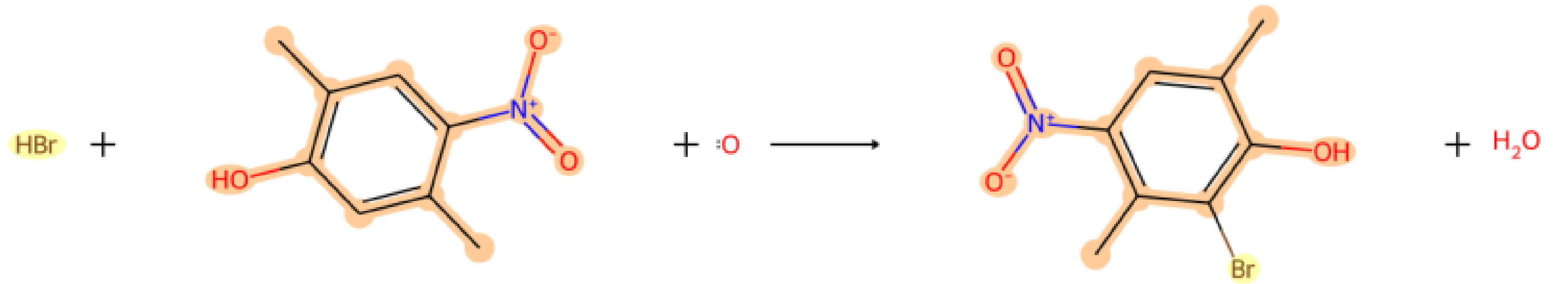

Ground Truth golden_dataset_467

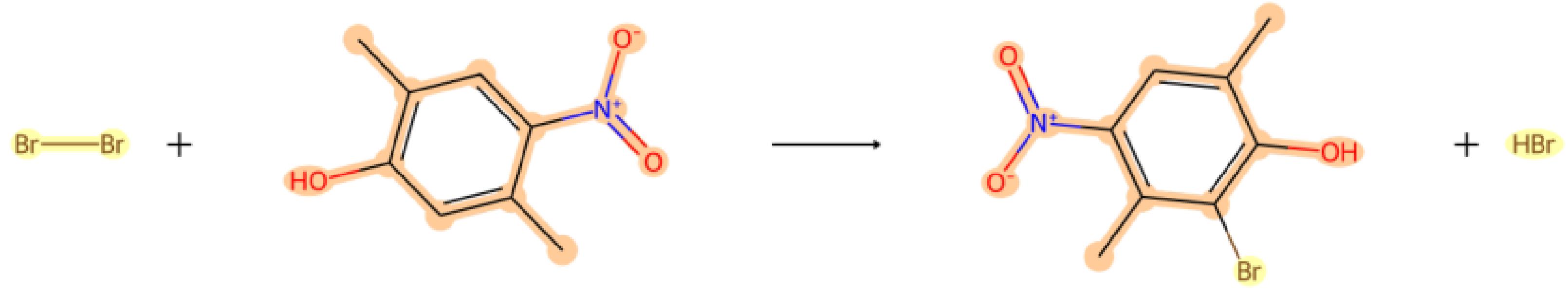

Original Reaction golden_dataset_468

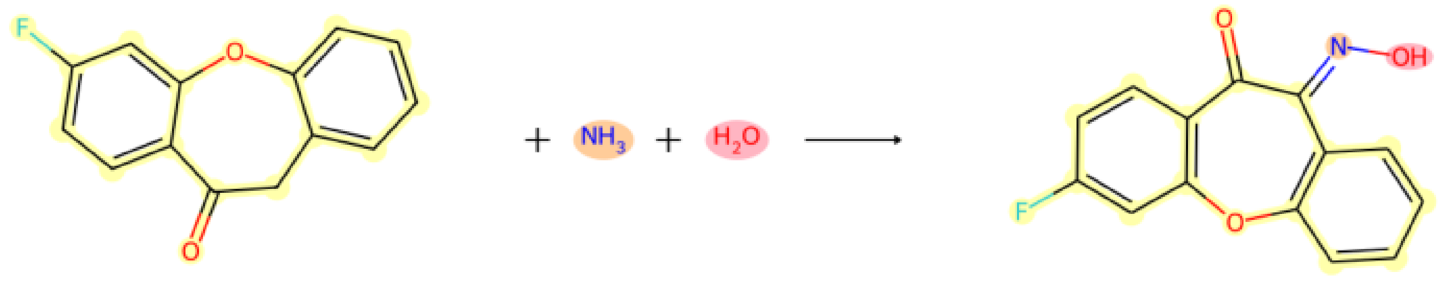

Imputed Reaction golden_dataset_468

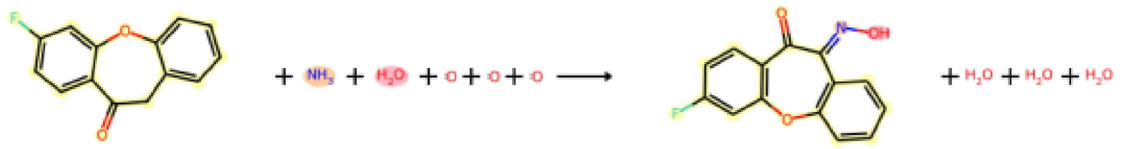

Ground Truth golden_dataset_468

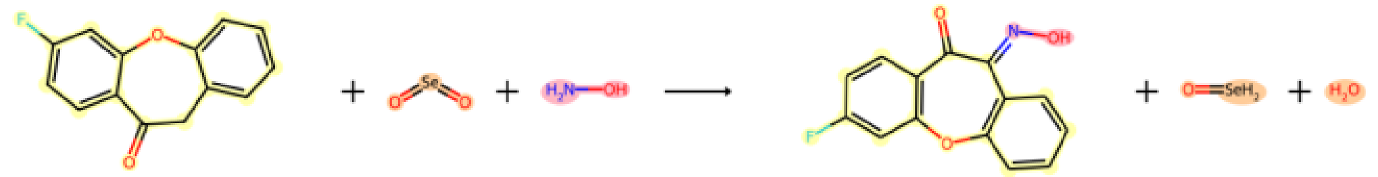

Original Reaction golden_dataset_475

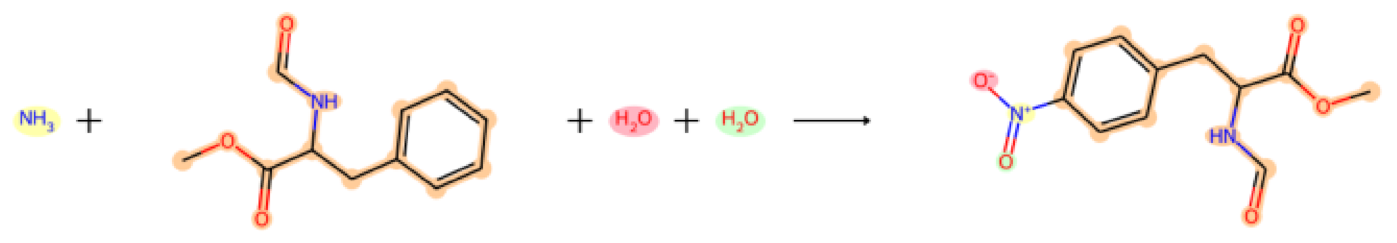

Imputed Reaction golden_dataset_475

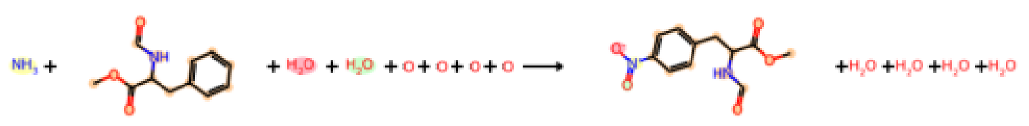

Ground Truth golden_dataset_475

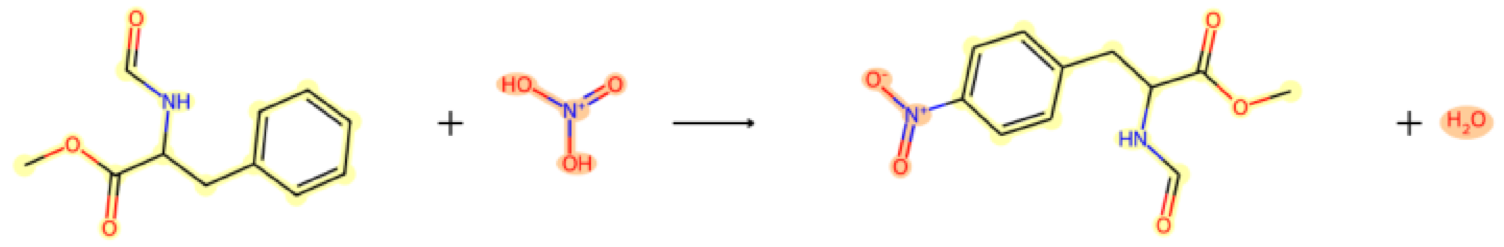

Original Reaction golden_dataset_517

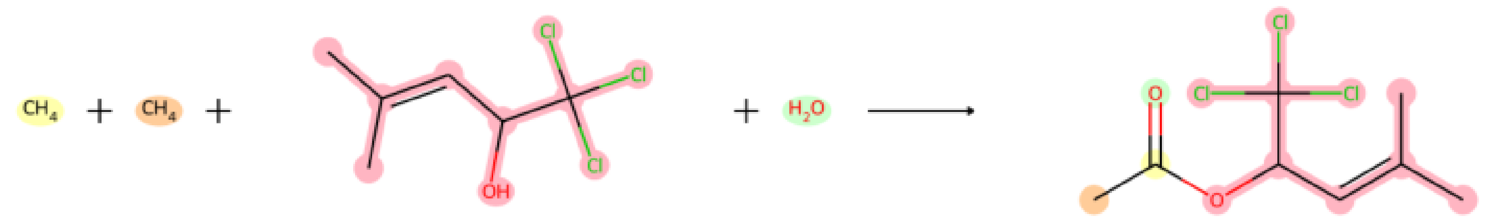

Imputed Reaction golden_dataset_517

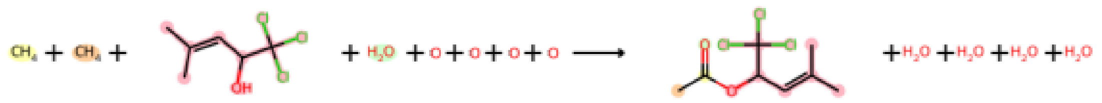

Ground Truth golden_dataset_517

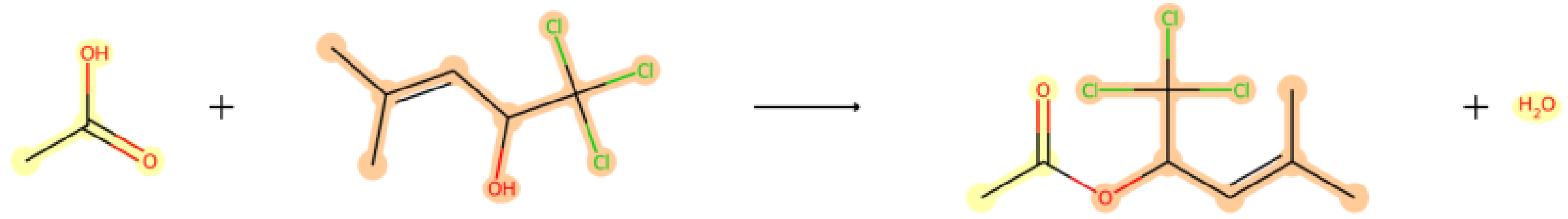

Original Reaction golden_dataset_521

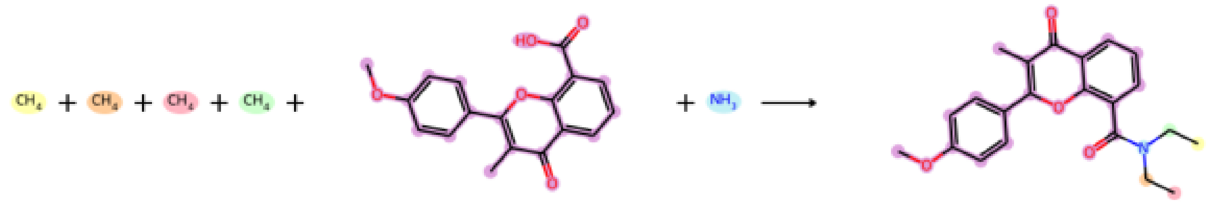

Imputed Reaction golden_dataset_521

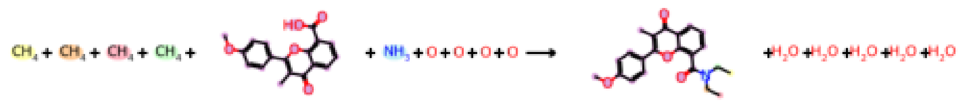

Ground Truth golden_dataset_521

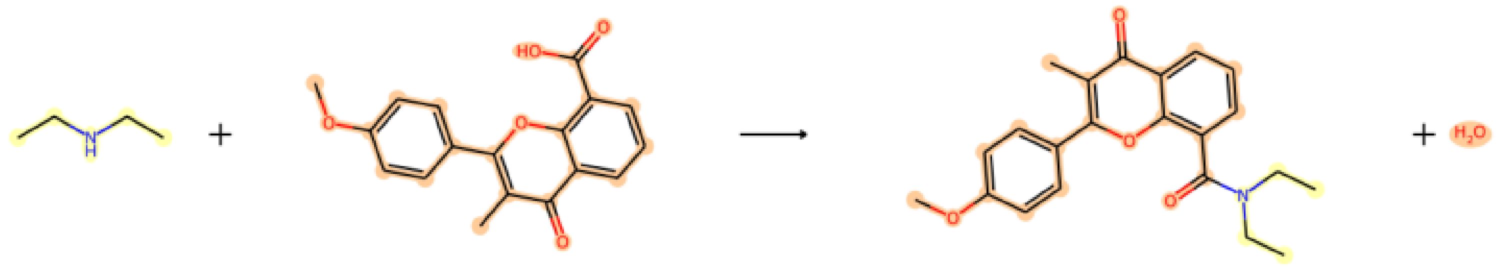

Original Reaction golden_dataset_549

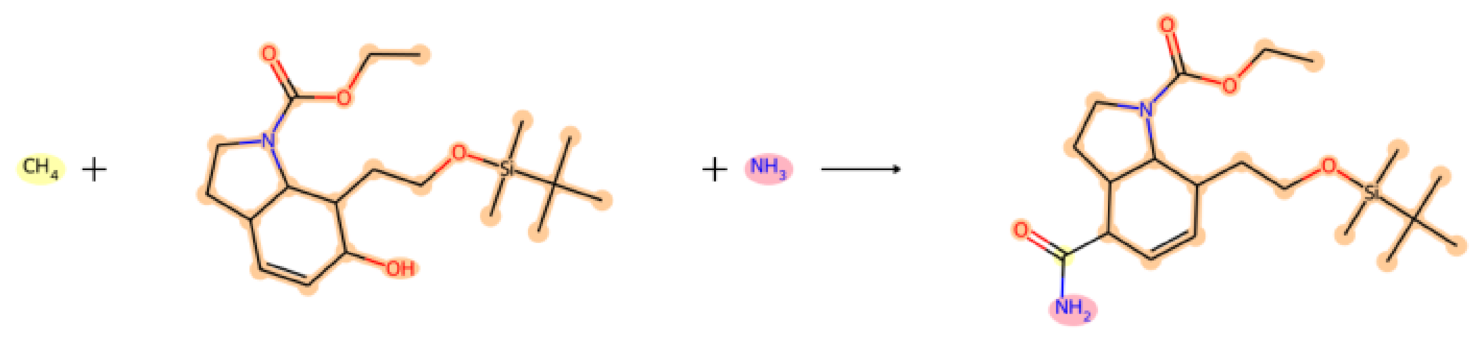

Imputed Reaction golden_dataset_549

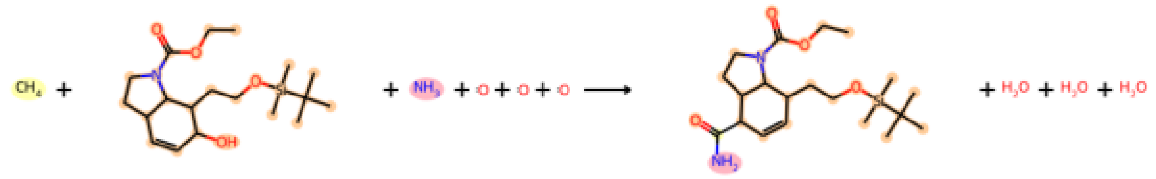

Ground Truth golden_dataset_549

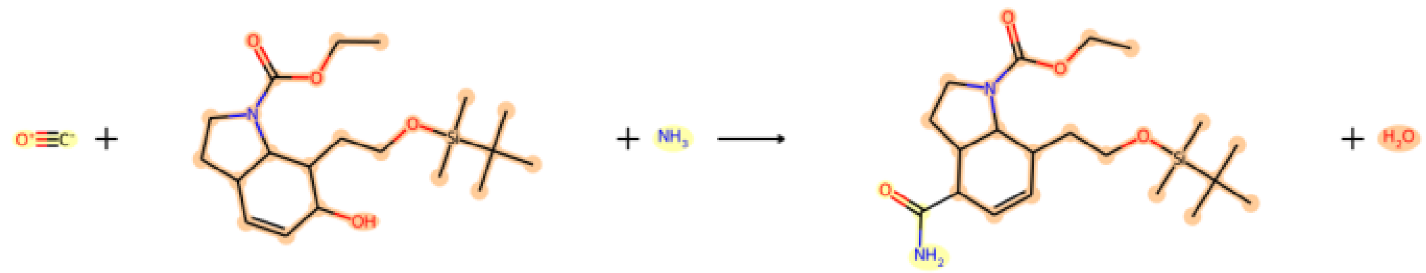

Original Reaction golden_dataset_730

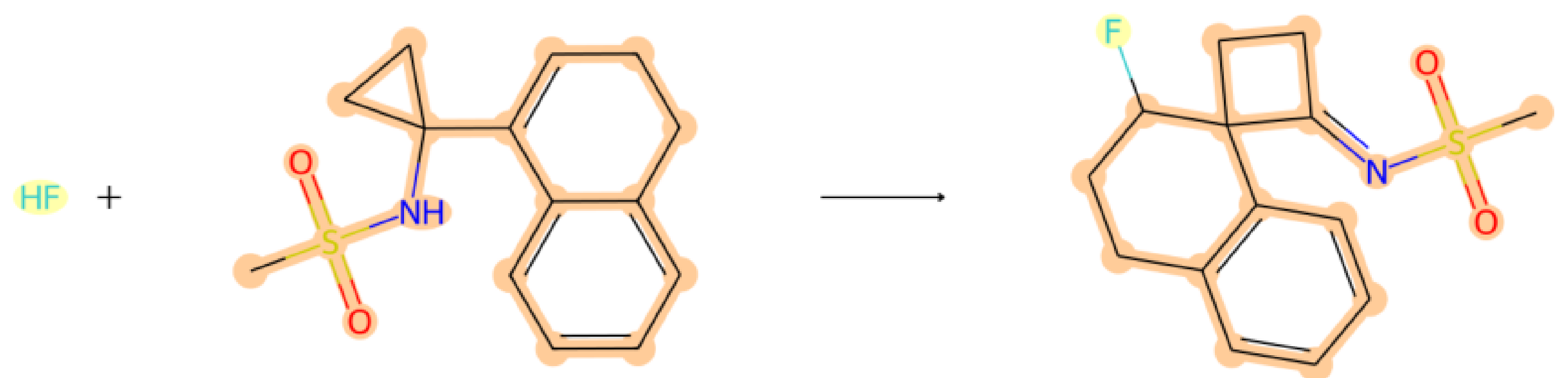

Imputed Reaction golden_dataset_730

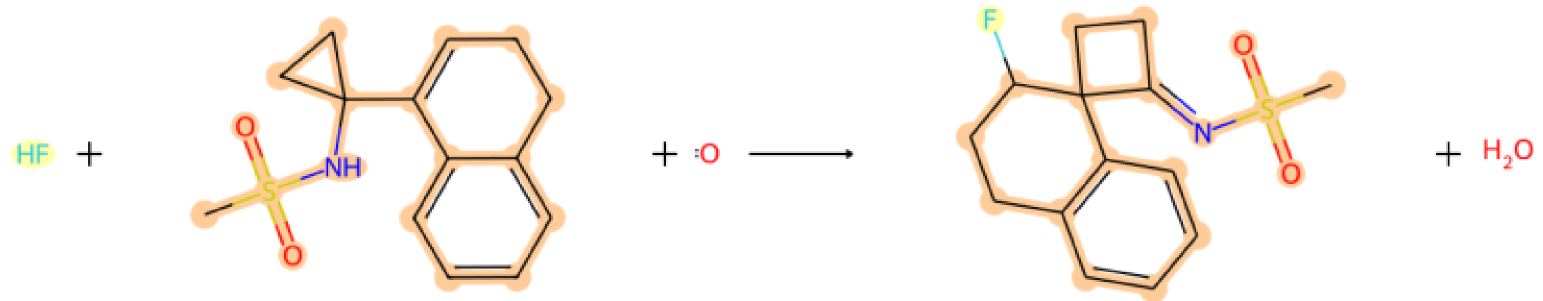

Ground Truth golden_dataset_730

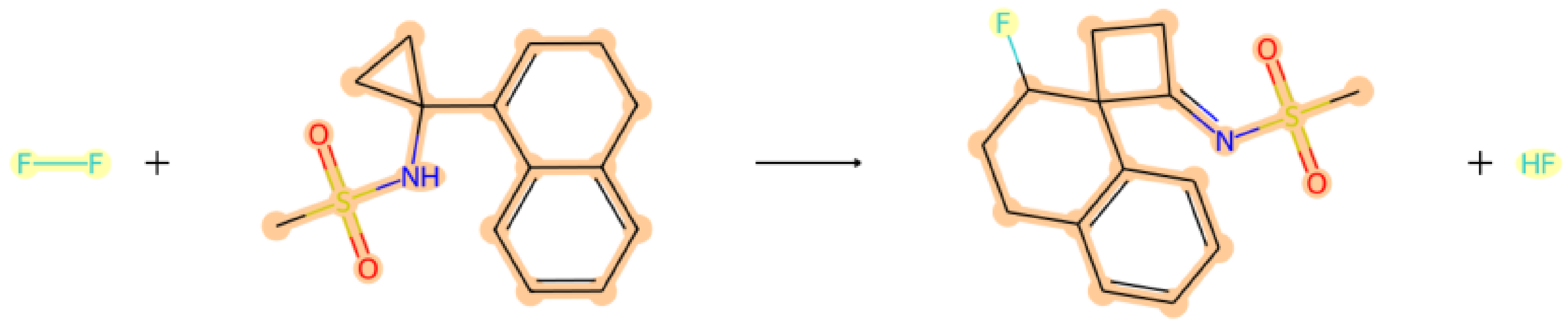

Original Reaction golden_dataset_752

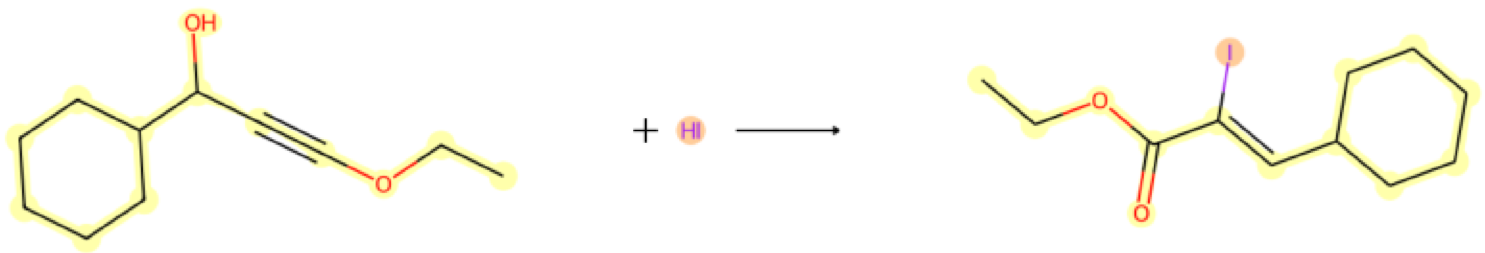

Imputed Reaction golden_dataset_752

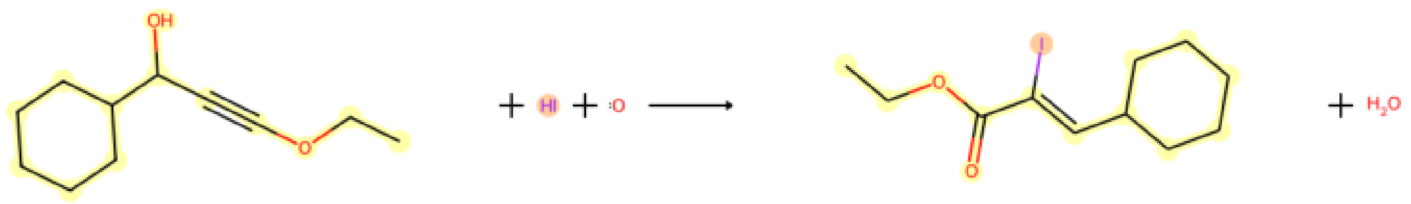

Ground Truth golden_dataset_752

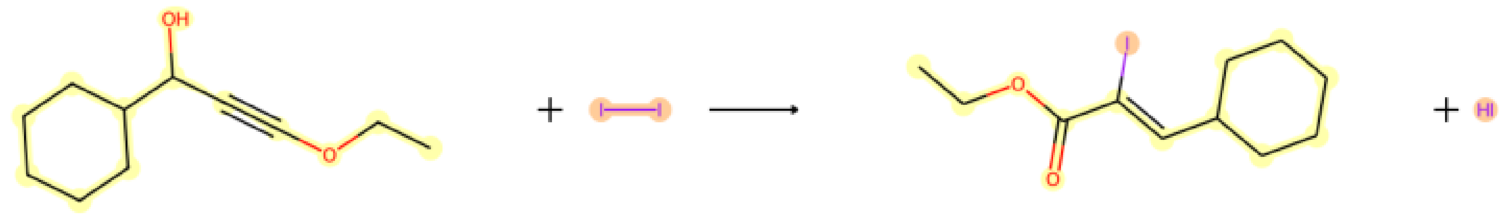

Original Reaction golden_dataset_754

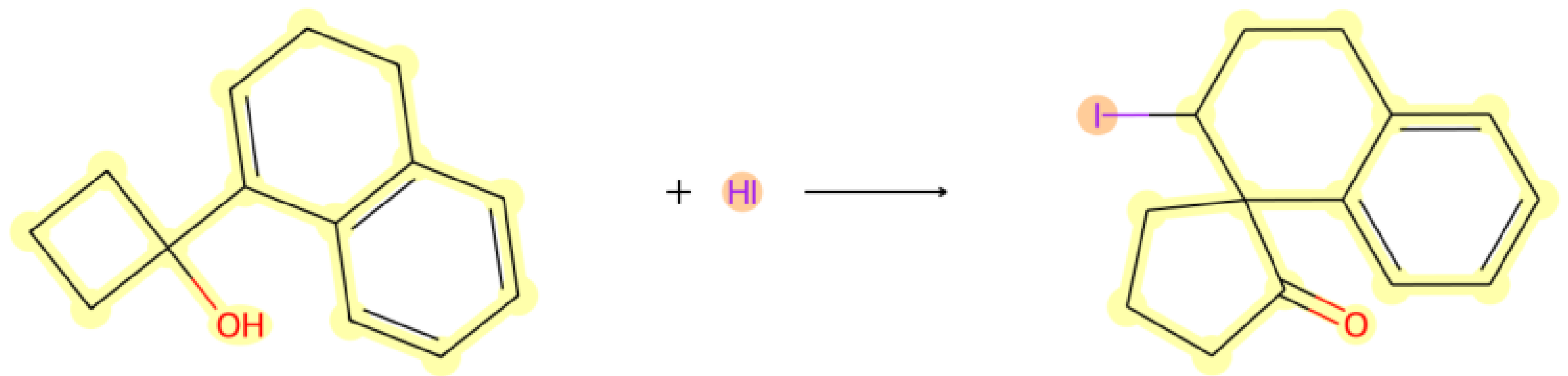

Imputed Reaction golden_dataset_754

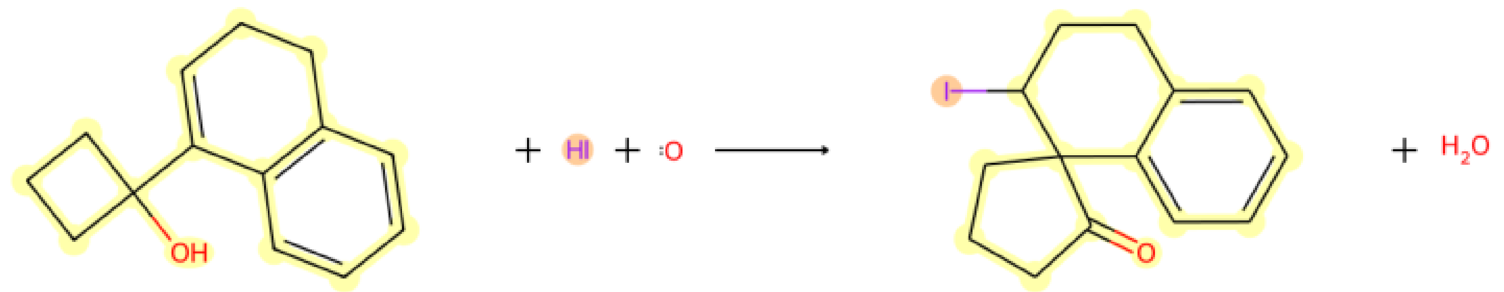

Ground Truth golden_dataset_754

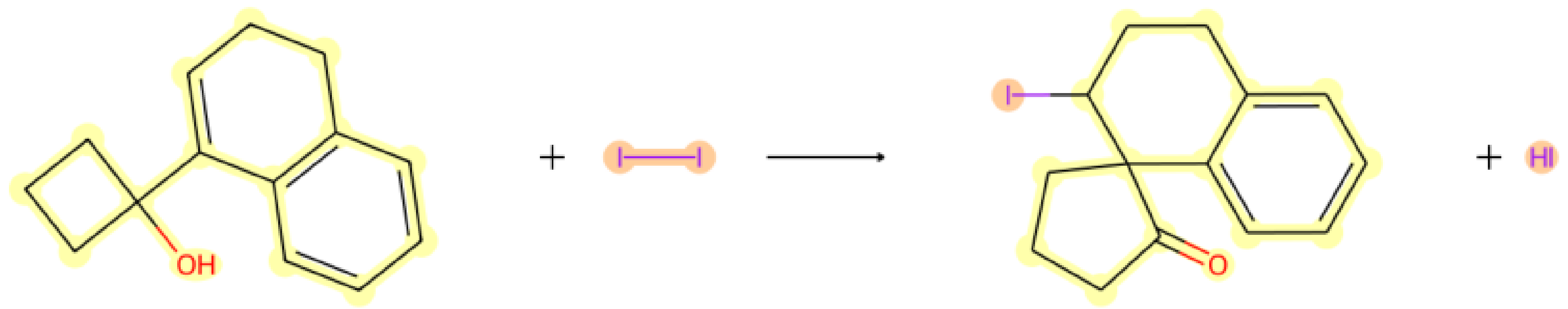

Original Reaction golden_dataset_913

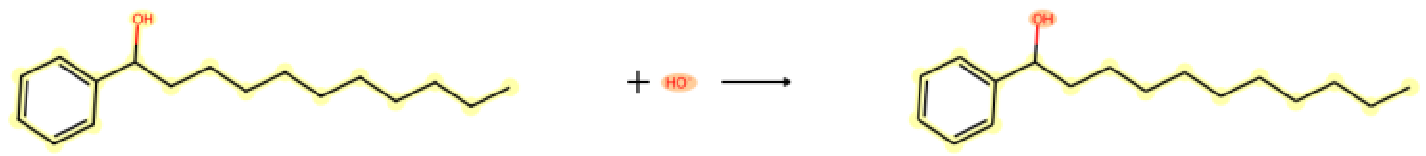

Imputed Reaction golden_dataset_913

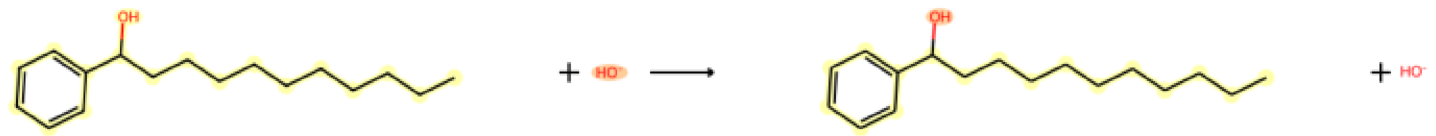

Ground Truth golden_dataset_913

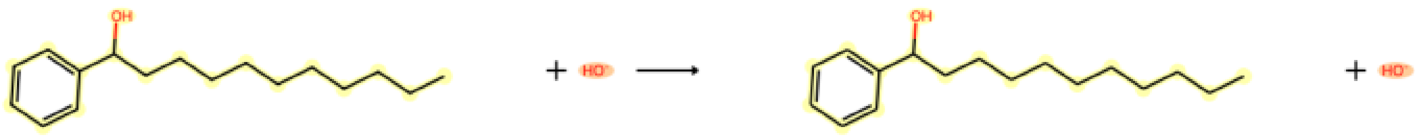

Original Reaction golden_dataset_936

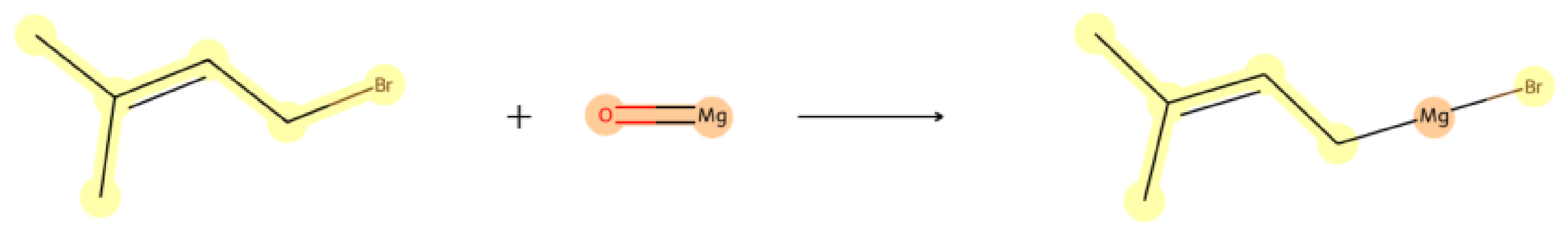

Imputed Reaction golden_dataset_936

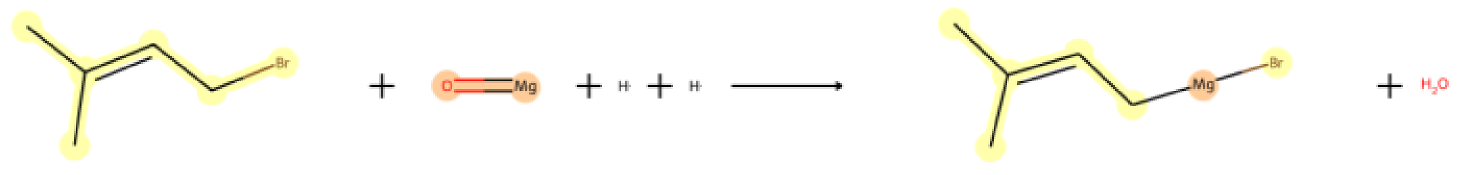

Ground Truth golden_dataset_936

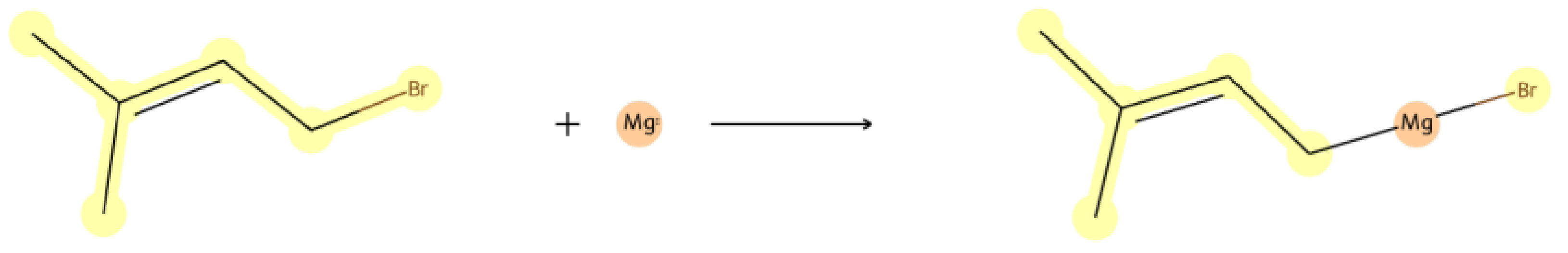

Original Reaction golden_dataset_1004

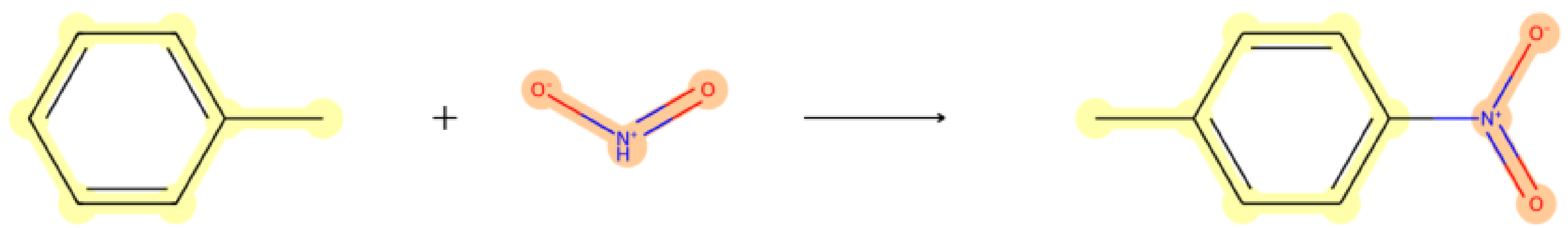

Imputed Reaction golden_dataset_1004

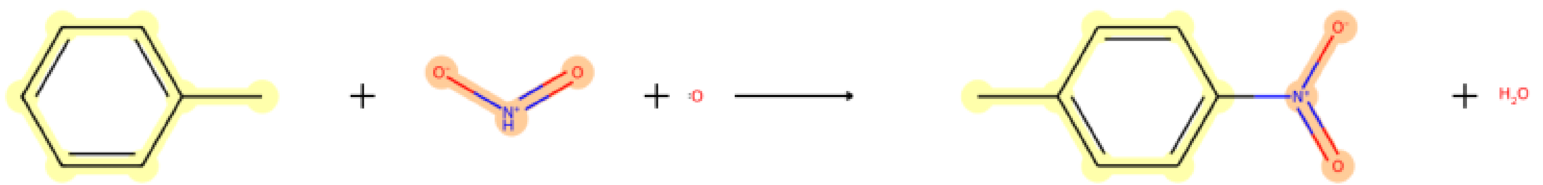

Ground Truth golden_dataset_1004

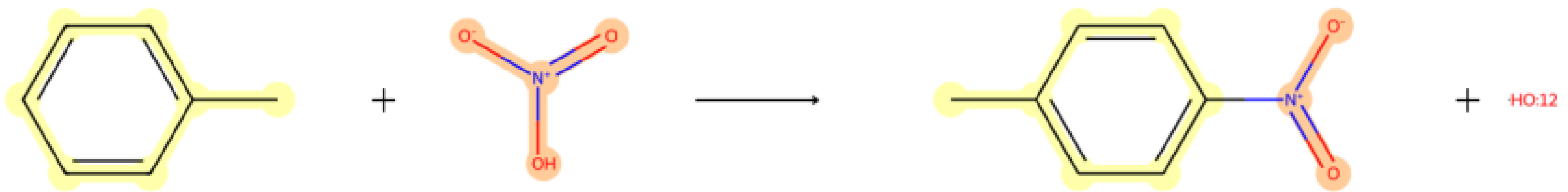

Original Reaction golden_dataset_1443

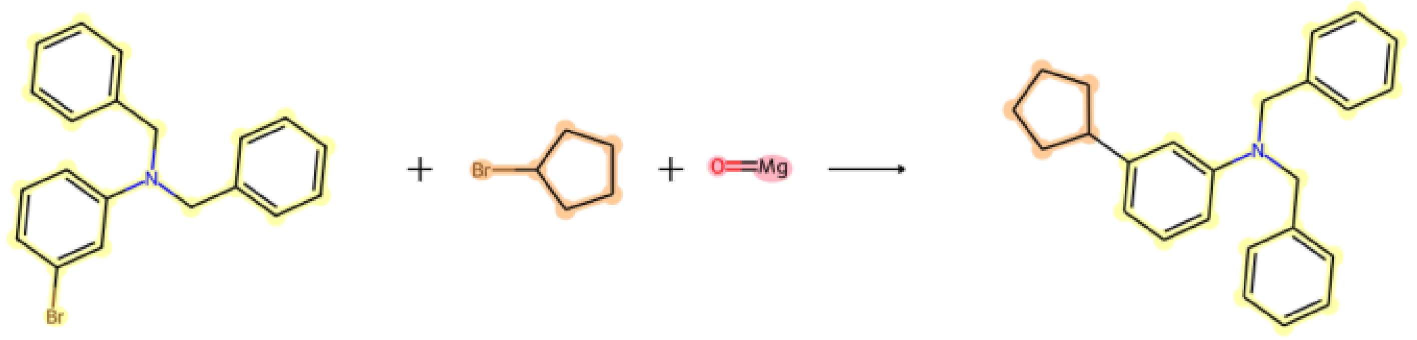

Imputed Reaction golden_dataset_1443

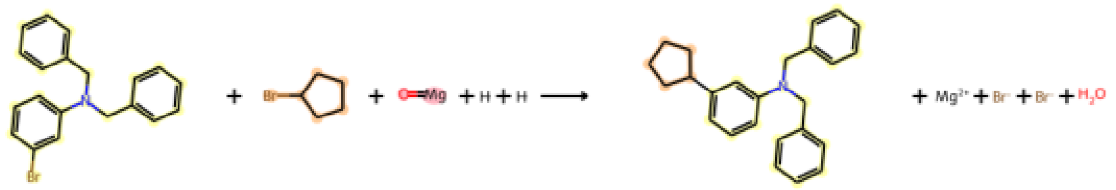

Ground Truth golden_dataset_1443

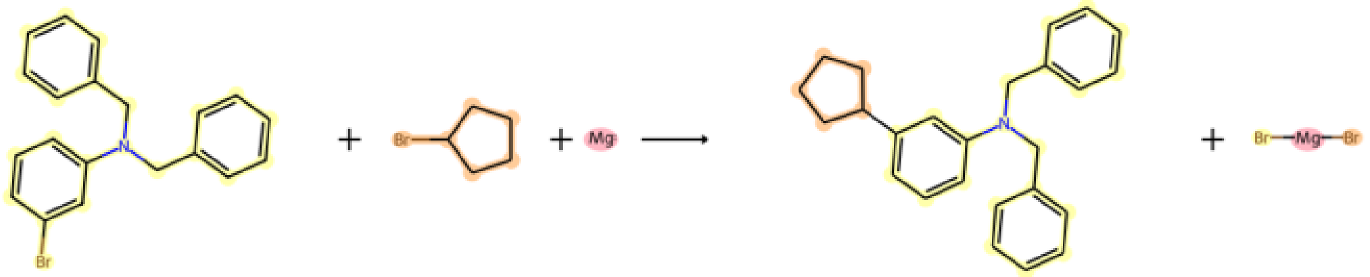

Supplement: Supplementary file 2 — Supplementary Material 2. [file 13321_2024_875_MOESM2_ESM.pdf]
